# Multi-Omics Insights into Gingivitis from a Clinical Trial: Understanding the Role of Bacterial and Host Factors

**DOI:** 10.3390/microorganisms13102371

**Published:** 2025-10-15

**Authors:** Niranjan Ramji, Ping Hu, Alejandra Muñoz Bodnar, Camila Pereira Braga, John Snowball, Dionne Swift, Hao Ye, Sancai Xie, Rachel Trenner, Malgorzata Klukowska, Eva Schneiderman, Aaron R. Biesbrock

**Affiliations:** 1 Oral Care, Global R&D, The Procter & Gamble Company, Mason, OH 45040, USA; munozbodnar.a@pg.com (A.M.B.); ye.h@pg.com (H.Y.); trenner.ra@pg.com (R.T.); klukowska.m@pg.com (M.K.); schneiderman.e@pg.com (E.S.); biesbrock.ar@pg.com (A.R.B.); 2 Discovery & Innovation Platforms, Corporate Function R&D, The Procter & Gamble Company, Mason, OH 45040, USA; hu.p@pg.com (P.H.); pereirabraga.c.1@pg.com (C.P.B.); snowball.jm@pg.com (J.S.); swift.dp@pg.com (D.S.); xie.s@pg.com (S.X.)

**Keywords:** microbiome, periodontal disease, stannous fluoride, proteomics, single cell, systematic effect

## Abstract

Poor oral health is a neglected epidemic, potentially contributing to systemic health issues. We employed a multi-omics approach to investigate the biological changes associated with gingivitis and the effects of stannous fluoride (SnF_2_) dentifrice on microbial composition and salivary proteomics in an eight-week clinical trial involving 39 participants categorized as high (*n* = 20) and low bleeders (*n* = 19). Baseline assessments revealed significant microbial dysbiosis in high bleeders, characterized by a higher abundance of *Porphyromonas* and *Fusobacterium*, alongside compromised epithelial barriers and increased inflammation. Following SnF_2_ treatment, a substantial reduction in these bacteria, and an increase in *Rothia* and *Haemophulis*, were observed, correlating with improved clinical measures, including reduced bleeding and inflammation indices. In total, 80 proteins (including pro-inflammatory cytokines, alarmin keratins, and matrix metalloproteinases) showed a significant reduction in high bleeders after treatment, with 29 overlapping the disease biomarkers in the plasma atlas, supporting the role of SnF_2_ in mitigating oxidative stress and enhancing epithelial integrity. Furthermore, SnF_2_ treatment significantly reduced collagen degradation, suggesting the preservation of tissue integrity. These findings highlight that SnF_2_ not only improves local oral health but may also benefit systemic health, showcasing the value of a multi-omics approach in understanding the interconnections among oral microbiota, inflammatory responses, and systemic health outcomes.

## 1. Introduction

The oral cavity hosts diverse microbial communities that are vital for local and systemic health. Maintaining the balance of these populations is crucial [1]; dysbiosis can lead to oral diseases like gingivitis and periodontitis, causing immune responses, tissue damage, and tooth loss [2]. Key bacteria include anaerobic, gram-negative pathogens, notably the “red complex” (*Porphyromonas gingivalis*, *Tannerella forsythia*, *Treponema denticola*) and “orange complex” species (*Fusobacterium nucleatum*, *Prevotella intermedia*, *Campylobacter rectus*) [3,4,5,6,7,8]. *P. gingivalis*, a significant keystone pathogen, drives dysbiosis and periodontal disease progression even in low abundance [9]. Oral biofilm formation begins with early colonizers like *Streptococcus*, which modify the environment and attract later colonizers, such as *Fusobacterium* and *Prevotella*. *F. nucleatum* serves as a bridge between early colonizers and the red complex, creating anaerobic niches that enable disease progression [10].

Emerging evidence indicates that periodontal diseases have significant implications for systemic health, affecting conditions like cardiovascular disease (CVD) [11], Alzheimer’s disease [12,13], diabetes [13], and chronic kidney disease (CKD) [14]. Chronic inflammation in periodontal tissues can cause chronic low-level bacteremia [15], which can elevate systemic biomarkers associated with atherosclerosis [16], increasing the risk of heart attacks and strokes. Additionally, periodontal bacteria and endotoxins may enter the bloodstream, potentially exacerbate neurodegenerative diseases [17,18,19] and influencing insulin resistance in patients with diabetes. Effective periodontal treatment has been shown to improve health outcomes, including blood glucose levels in diabetics and reduced inflammation, emphasizing the need for integrated oral healthcare. Overall, oral infections can lead to systemic diseases through mechanisms such as bacteria entering the bloodstream and causing inflammation and tissue damage.

Multi-omics, the integrated analysis of genomics, transcriptomics, proteomics, and metabolomics, has become a transformative approach in biomedical research, particularly for complex diseases like oral conditions [20,21]. Metagenomics has extensively studied the oral microbiome in relation to periodontal disease and dental caries, highlighting significant links between microbial diversity, genetic variations, and disease states [22,23]. Proteomics aids in identifying protein biomarkers in oral fluids and tissues, including saliva and gingival crevicular fluid [24,25]. Recent advancements in single-cell RNA sequencing (scRNA-seq) allow for the detailed exploration of gene expression in individual cells, enabling the study of cellular heterogeneity and rare cell types in oral tissues [26]. Together with metagenomics, these technologies shed light on the complex relationships between the oral microbiome and dental health. Public databases, like the UK Biobank, enhance our understanding of disease connections through multi-omics analysis. A recent study mapped the plasma proteome in over 53,000 adults, identifying numerous protein-disease associations and potential biomarkers for precision medicine [27]. By analyzing these datasets, researchers can uncover novel associations between oral health and systemic conditions, providing a comprehensive understanding of the factors influencing the etiology and treatment of oral diseases [28].

Recent studies have examined various oral care ingredients for their antimicrobial properties, with stannous fluoride (SnF_2_) identified as a particularly effective agent. SnF_2_ modulates the oral microbiome, protects tissues by maintaining barrier integrity, and may influence systemic health [29,30,31]. Research shows that SnF_2_ inhibits the virulence of oral pathogens by binding to lipopolysaccharides and blocking their interaction with Toll-like receptors [32], thereby reducing inflammation and oxidative stress in the oral cavity [33]. Clinical studies indicate that SnF_2_ significantly decreases gingival inflammation and enhances antioxidant capacity. Additionally, it may lower systemic inflammation markers in individuals with high CRP levels and reduce cardiovascular disease risks by decreasing oxidized low-density lipoprotein levels [33]. However, the precise molecular mechanisms by which these ingredients affect the oral microbiome and interact with host tissues remain unclear. While recent transcriptomic studies have begun to explore how oral care products influence the gene expression of key pathogens, many connections within this complex interplay require further investigation [34].

This research aimed to determine the relationship of 0.454% SnF_2_ dentifrice treatment with oral microbiome composition and host biomarker response in patients with gingivitis (high bleeders) versus a control healthy patient population (low bleeders). Using samples from a previous clinical trial [33], we employed a multi-omics approach that integrates data from microbial composition, proteomic profiles, and in vitro assays. We also utilized single-cell and plasma proteomics datasets to clarify interactions among oral biomarkers and their potential links to overall health. The null hypothesis was that SnF_2_ therapy does not differentially affect the oral microbiome composition and host biomarker response in gingivitis patient populations relative to healthy patient populations.

## 2. Materials and Methods

### 2.1. Experimental Design and Sample Collection

This study was a single-center, single-treatment clinical study designed to evaluate the effects of 0.454% SnF_2_ dentifrice exposure on the oral microbiome composition and host biomarker response, comparing treatment responses in the experimental group (gingivitis high-bleeder population) with the control group (healthy low-bleeder population). As described in the clinical publication [33], the study involved 39 adult volunteers, divided into two groups: 19 healthy subjects with up to three bleeding sites and pocket depths ≤ 2 mm (low bleeders) and 20 unhealthy subjects with more than 20 bleeding sites and at least three pockets between 3 mm and 4 mm deep (high bleeders). Participants were 18 years or older, provided written informed consent, had at least 16 natural teeth, and were in good general health. Exclusions included pregnancy, inability to comply with study procedures, severe periodontal disease, recent dental prophylaxis, antibiotic use within two weeks of sampling, and any condition interfering with safe study completion. All participants used 0.454% SnF_2_ toothpaste (Crest^®^ Pro-Health Sensitive and Enamel Shield, Cincinnati, OH, USA) twice daily for eight weeks. They brushed for one minute using the provided soft manual toothbrush (Oral-B^®^ Indicator, Cincinnati, OH, USA). Subjects received an oral examination along with Modified Gingival Index (MGI) and Gingival Bleeding Index (GBI) exams at each visit. Pocket depth was measured at the screening visit. Baseline demographic and clinical characteristics of participants are presented in Table S1. Unstimulated saliva samples (up to 3 mL) were collected at baseline and Week 8, placed on dry ice immediately, and stored long term at −70 °C and analyzed for protein, enzymes, and metabolites. Gingival Crevicular Fluid (GCF) samples were collected from the buccal surfaces of premolars and molars using periopaper strips as described earlier [33]. GCF samples were pooled into a tube with a buffer and stored on dry ice for the collagenase assay.

### 2.2. Microbiome Sample Collection and Analysis

Subjects were reminded to refrain from brushing their teeth, eating, drinking (small sips of water were allowed up to 45 min prior to the visit), flossing, chewing gum, using breath mints, and using tobacco after their evening brushing and before the morning of the sample collection visit. Supragingival plaque samples were collected at baseline and four weeks and then were transferred to pre-labeled tubes with 200 μL of buffer and stored on dry ice and frozen at −70 °C until transferred to a P&G laboratory. DNA samples were processed and sequenced using the ZymoBIOMICS^®^ 16S rRNA Gene Sequencing Service (Zymo Research, Irvine, CA, USA) for 16s V3-V4 region. Sequence data were analyzed using the EZBiom 16s Analysis software PKSSU4.0 [35] for taxonomy classification and gene function identification. Cycle threshold (Ct) values from the real-time PCR amplification were used to filter samples, and samples with Ct values greater than 30 were excluded from all statistical analyses involving microbiome data. All statistical analyses were performed using R software (version 4.1.3). Differences in the microbial community between groups were evaluated using both univariate and multivariate analyses. For univariate analyses of alpha diversity and individual taxa, the Kruskal–Wallis test was performed, followed by pairwise Wilcoxon Rank Sum tests to compare independent samples. For dependent or paired samples, the Friedman test was utilized, followed by pairwise Wilcoxon Signed Rank tests to assess group differences. In multivariate community analyses, differences in group-based beta diversity, as determined using Bray–Curtis dissimilarity, were assessed using permutational multivariate analysis of variance (PERMANOVA), a non-parametric method implemented through the R package ‘adonis2’. Nonmetric multidimensional scaling (NMDS) analyses were conducted to visualize the community clustering of samples based on Bray–Curtis similarities. Both unadjusted and adjusted *p*-values, using the Benjamini–Hochberg false discovery rate correction, were reported to account for multiple comparisons.

### 2.3. Saliva Proteomics

A volume corresponding to 50 μg of protein was precipitated with four volumes of cold acetone and incubated at −20 °C for one hour. Following centrifugation at 14,000× *g* for 10 min, the supernatant was discarded, and the resulting pellets were air-dried and resuspended in 20 μL of denaturation buffer (50 mM ammonium bicarbonate, pH 8.5; 10 mM TCEP; 5% sodium deoxycholate). Samples were incubated at 60 °C for 10 min to ensure complete solubilization, then alkylated by adding 5 μL of 100 mM iodoacetamide and incubated for 1 h at room temperature in the dark. Subsequently, 175 μL of 50 mM ammonium bicarbonate (pH 8.0) was added, followed by 2 μL of trypsin (1 μg/μL), and samples were digested overnight at 37 °C in the dark. Digestion was quenched, and sodium deoxycholate was removed by adding 10 μL of 10% trifluoroacetic acid, incubating for 30 min, and centrifuging at 15,000× *g* for 10 min. The resulting supernatants were collected for LC-MS/MS analysis. MS raw files were processed with MaxQuant. MS/MS spectra were matched to in silico-derived tryptic peptide fragment mass values from the Uniprot human database UP000005640. Label-free quantification (LFQ) was performed using classic normalization. After MaxQuant processing, data were filtered, and proteins that were present in at least 50% of the samples were considered valid. After data cleaning and log2 transformation of the intensities, missing values were imputed at the protein level using random draws from a Gaussian distribution centered on the minimum value (MinProb method). For each protein, two sample tests were used to compare low and high bleeders at baseline. For high bleeders and each protein, a linear mixed model with terms for time (BL and Week 8) and a random subject was used to compare baseline and Week 8 visits. Volcano plots of differentially regulated proteins and various graphs were generated using R. A protein was considered differentially expressed if it exhibited a fold change (FC) ≥ 1.5 and *p*-value ≤ 0.05.

### 2.4. Single Cell Data Integration

Single-cell data from previously published control buccal and gingival biopsies were downloaded, reprocessed, and annotated [36]. In short, individual biopsy gene expression matrices were used to create a Seurat single-cell object before high-quality cells were isolated (nFeature_RNA > 200 & percent.mt < 15 & nFeature_RNA < 5000) and preliminarily clustered [37]. After initial clustering, all control buccal and gingival samples were merged using anchor integration, re-clustered, and annotated using markers previously reported. Marker genes had a minimal percentage (min.pc) greater than 0.25 and a log fold-change threshold greater than 1. For the identification of immune subtypes, major immune clusters were subsetted out, re-clustered, and re-annotated. Ucell was used to determine if any genes corresponding to significant protein changes were enriched in any specific cell type [38]. Ucell graphs were generated using Prism and plotted with mean and SD. A two-way ANOVA followed by a Kruskal–Wallis test were used to determine clusters with significantly higher Ucell scores than all other clusters, *q* < 0.01 (version 10.0.0 for Windows, GraphPad Software, Boston, MA, USA, www.graphpad.com). Protein changes corresponding to single-cell marker genes were visualized in a Z-score normalized heatmap of protein log2(normalized intensities). Functional enrichment analyses of specific protein changes were performed using ToppFun [39].

### 2.5. Collagen Degradation Analysis

For performing Human Crosslinked C-terminal Telopeptide of Type 1 Collagen (ICTP), a MyBiosource Cat#MBC040005 Quantitative Sandwich ELISA was utilized (MyBioSource, San Diego, CA, USA). Collected GCF samples were allowed to thaw to room temperature prior to centrifugation. Samples were centrifuged for 20 min at 1000× *g*. Post centrifugation, 20 μL of GCF solution were diluted with 100 μL of sample diluent. The execution of standards, samples, and blanks was performed following the kit directions.

### 2.6. Disease Association Analysis

Plasma protein biomarkers associated with a variety of diseases with a *p*-value less than 0.05 were selected from the “Atlas of the Plasma Proteome in Health and Disease” [27]. From the baseline data, proteins with significant differences between healthy and unhealthy groups (*p* < 0.05) that showed the same direction were identified as disease plasma proteomic markers and counted, then displayed on a bar plot. The percentage of these overlapped proteins to the total plasma proteomics disease markers was plotted on the line to reflect that different diseases might have different numbers of disease markers in the atlas database. After the eight-week treatment, proteins that showed opposite direction changes (with a *p*-value ≤ 0.05) to the disease markers were counted, and the percentage of these proteins to the total number of the plasma protein biomarkers was calculated and plotted as a bar or line plot.

## 3. Results

### 3.1. Supragingival Plaque Microbiome Differs in High Bleeder vs. Low Bleeder at Baseline

**Figure 1:**
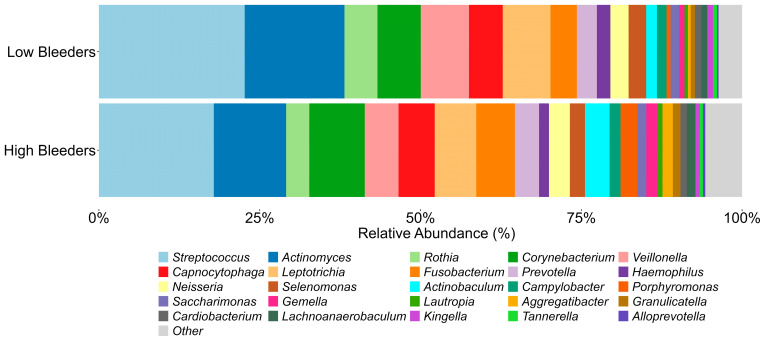
Oral microbiome components from high bleeders vs. low bleeders. The top 25 genera detected from the supragingival plaque of high bleeders and low bleeders at baseline.

The composition of supragingival plaque microbiota exhibits significant differences between low and high bleeders (Figure 1, Table S2). Several oral bacterial genera associated with gingivitis, including *Porphyromonas* (*p* < 0.01) and *Alloprevotella* (*p* = 0.02), show higher relative abundances in high bleeders, with *p*-values indicating significance at an α level of 0.05. Additionally, while the *p*-value for *Fusobacterium* (*p* = 0.089) does not reach conventional significance at 0.05, it demonstrates a directional difference.

### 3.2. Saliva Proteomics and Single-Cell Integration Analyses Reveal Proteins Differentially Expressed at Baseline in High Bleeders Compared with Low Bleeders

**Figure 2:**
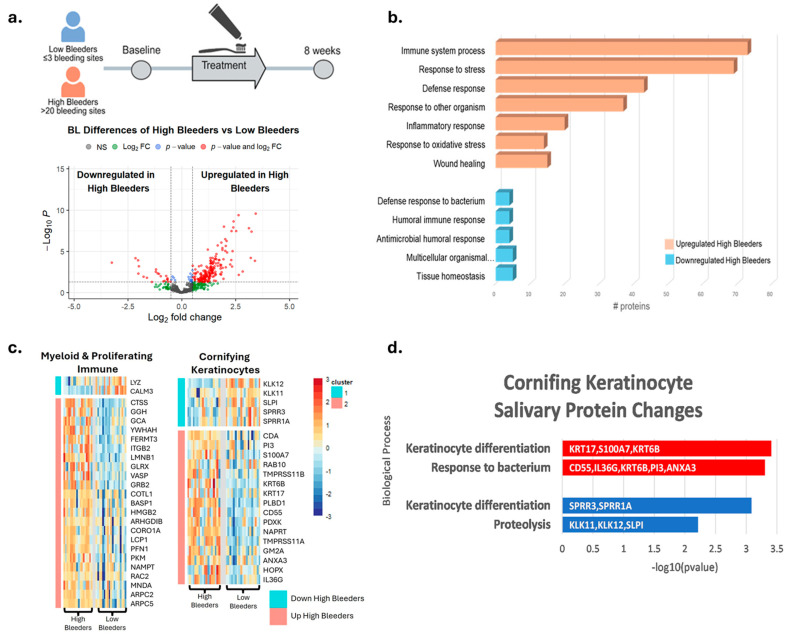
Proteomics and single-cell integration analyses reveal differentially expressed proteins at baseline in high bleeders compared with low bleeders. (**a**) Proteomics study design: Saliva samples were collected from high and low bleeders at baseline and after eight weeks of toothpaste treatment. The volcano plot illustrates the differentially expressed proteins between high and low bleeders at baseline, using a fold change (FC) ≥ 1.5 and a *p*-value ≤ 0.05 as criteria. The x axis represents log2 fold change. The y axis represents −log10 (*p* value). Grey dots represent proteins without significant changes (NS: not significant); red dots indicate proteins with a significant fold change (*p* ≤ 0.05 and FC ≥ 1.5); blue dots represent proteins with significant changes (*p* ≤ 0.05) but with FC < 1.5; green dots signify proteins with FC ≥ 1.5 but not significant (*p* > 0.05). (**b**) Enrichment analysis of the differentially expressed proteins highlights the biological processes associated with the proteins that are upregulated and downregulated in high bleeders. (**c**) Single-cell overlay of protein changes reveals specific markers of myeloid and cornifying keratinocytes that are altered in high bleeders. (**d**) Functional enrichment analyses of protein changes associated with markers of the cornifying keratinocyte population are linked to altered keratinocyte differentiation and bacterial response. Red indicates upregulation, while blue denotes downregulation.

For proteomics, saliva samples were collected at baseline and after eight weeks of treatment with SnF_2_ toothpaste in both high and low bleeders (Figure 2a). A total of 192 proteins were identified as significantly different between high and low bleeders, with 173 upregulated and 19 downregulated in high bleeders at baseline (Figure 2a, Supplementary Table S3). To elucidate the underlying biological mechanisms, an enrichment analysis was performed (Figure 2b). In high bleeders, processes such as tissue homeostasis, antimicrobial humoral response, and defense response to bacteria were downregulated. In contrast, processes related to immune system activity, response to stress, inflammatory response, and response to oxidative stress were upregulated.

Specifically, increased oxidative stress is evidenced by the upregulation of antioxidant enzymes, such as SOD2 and GSR, in high bleeders, suggesting a compensatory response to elevated reactive oxygen species (ROS) production (Supplementary Tables S3 and S4) and subsequent cellular damage. Inflammation is marked by increased levels of inflammatory cytokines and enzymes, including IL36G and matrix metalloproteinases (MMPs), which can promote tissue inflammation and degradation. Proteins like CAMP serve dual roles in both antimicrobial defense and the regulation of inflammation. The observed downregulation of processes related to tissue homeostasis and antimicrobial defense may further compromise the integrity of the oral mucosa, rendering it more susceptible to pathogenic insults and impairing healing. Collectively, these data highlight a complex network of dysregulated mechanisms in high bleeders, where the interplay between oxidative stress, inflammation, and immune response creates a pro-inflammatory environment that can facilitate tissue destruction and disease progression.

To help understand where the proteins detected in salivary samples may originate, publicly available single-cell transcriptional data were used to predict the most likely cells contributing to the changes in protein measured using mass spectrometry (Figure S1a). The protein changes detected when comparing high bleeders with low bleeders and proteins that changed after treatment were used to generate gene signatures. These gene signatures were then overlayed onto single-cell data using UCELL to generate an enrichment score for each signature in each cell and cell type. The genes that correspond to proteins that increased in high-bleeder salivary samples were significantly enriched in myeloid and proliferating immune cells, with decreased proteins being enriched in cornifying keratinocytes (Figure S1b). An overlay of the marker genes of these populations with protein changes in high bleeders revealed an increase in specific myeloid markers and an alteration of baseline cornifying keratinocyte markers (Figure 2c). To determine which specific immune population was likely accounting for this signal, all immune cells were extracted and re-clustered into unique immune sub-populations. At this resolution, the neutrophils, mDC, and proliferating immune cell clusters are enriched for the genes that correspond to the upregulated proteins in high-bleeder saliva, likely indicating that they are major contributors to protein induction (Figure S1c,d). Functional enrichment analyses of the cornifying keratinocyte-associated proteins up- or downregulated indicate alteration to keratinocyte differentiation and an active bacterial response (Figure 2d). Specifically, KRT17 and KRT6B, which are known as “Alarm Molecules” for epidermal barrier disruption [40], were increased. Coupled with the increase in S100A7, IL36G, and PI3, which are associated with bacterial response, these data indicate a compromised epidermal barrier and bacterial infection in high bleeders.

### 3.3. Saliva Proteomics Results and Their Association with Human Disease

**Figure 3:**
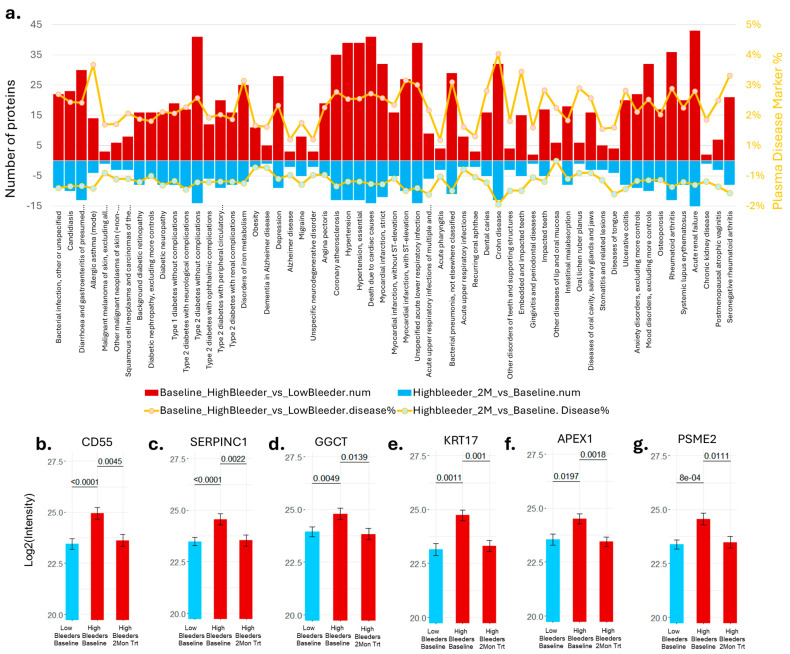
Proteomics results and their association with human disease. (**a**) Number of proteins that showed significant changes consistent with the plasma disease protein markers [27] at baseline were reported as the top red bar. The bottom blue bar shows the number of proteins in the opposite direction after eight weeks of treatment compared with the plasma disease marker. The ratio of the overlapped proteins to the total number of plasma disease markers was displayed in the orange line plot. (**b**) APEX1 protein intensity was detected in the supragingival plaques. (**c**) CD55 protein intensity was detected in the supragingival plaques. (**d**) GGCT protein intensity was detected in the supragingival plaques. (**e**) KRT17 protein intensity was detected in the supragingival plaques. (**f**) PSME2 protein intensity was detected in the supragingival plaques. (**g**) SERPINC1 protein intensity was detected in the supragingival plaques.

We compared the identified proteomic biomarkers with the human plasma disease biomarkers from the “Atlas of the Plasma Proteome in Health and Disease” [27]. In total, 192 proteins showed significant differences between high bleeders and low bleeders at baseline; 71 of those were disease biomarkers that overlapped with the plasma proteomics atlas. These 71 baseline gingivitis proteomic biomarkers correlated with biomarkers associated with a range of human diseases, including infectious diseases, cardiovascular conditions, diabetes, dementia, and depression. A subset is shown in Figure 3a. The overlapped oral protein biomarkers are roughly 1–4% of the disease biomarkers reported in the plasma proteomics atlas. We further revealed that, after eight weeks of treatment with SnF_2_ dentifrice, 80 proteins showed significant changes in high bleeders compared with baseline data. In total, 29 of them are referenced in the plasma atlas as disease biomarkers. The treatment-induced protein change indicates a shift from disease towards a healthier status. Overall, 25–40% of the overlapped disease biomarkers were altered after eight weeks of treatment in a “healthy” direction. This suggests that stannous treatment can positively impact these biomarkers. There are 18 proteins in all three datasets: ALDH1A1, APEX1 (Figure 3b), CD55 (Figure 3c), CHIT1, F2, GBP6, GGCT (Figure 3d), GM2A, KLK12, KRT17 (Figure 3e), MTPN, MYDGF, NAPRT, PI3, PSME2 (Figure 3f), SERPINC1 (Figure 3g), TMPRSS11B, and YWHAQ. It has been reported that increased plasma levels of APEX1, CD55, PSME2, and SERPINC1 are significantly associated with hypertension, and increased plasma levels of APEX1, CD55, GGCT, KRT17, PSME2, and SERPINC1 are associated with diabetes (Table S5) [27]. At baseline, the levels of these proteins were higher in high bleeders compared with low bleeders. After eight weeks of treatment, there was a decrease in protein levels from baseline, particularly in high bleeders.

### 3.4. Correlation of the Clinical Measurements, Proteomics, and Microbiome

**Figure 4:**
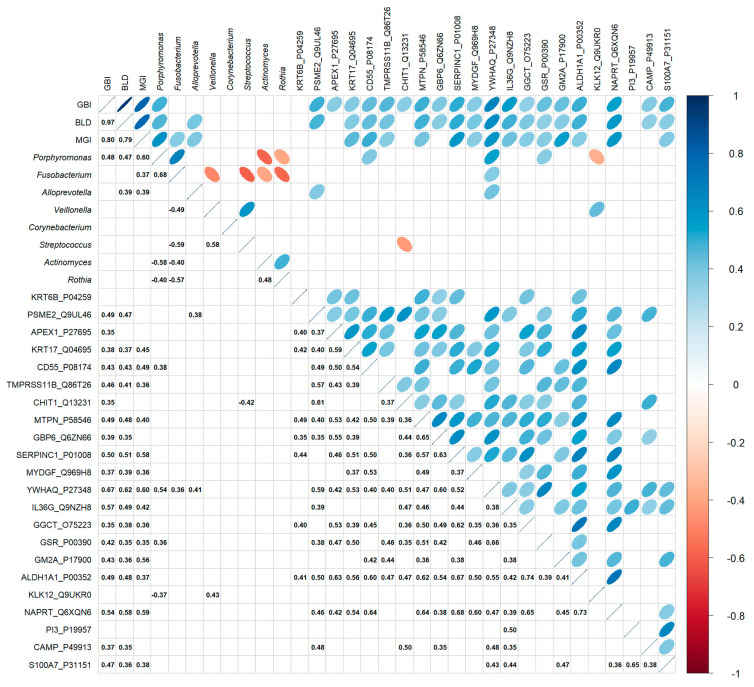
Correlation between the relative abundance of selected genera from supragingival plaques of all subjects, clinical measurements, and selected protein measurement at baseline. The various correlation comparisons are listed on the X and Y axes, with correlation directionality and magnitude indicated by the color and size of the ellipse (statistically significant correlations only). The bottom half of the graph shows the Spearman correlation coefficient with *p* ≤ 0.05.

*Alloprevotella*, *Porphyromonas*, and *Fusobacterium* positively correlated with clinical measurements (Figure 4). These three bacteria are also positively associated with YWHAQ (Alloprevotella–Spearman correlation coefficient CC = 0.41, *p* = 0.02; Porphyromonas–Spearman CC = 0.52, *p* = 0.001; Fusobacterium–Spearman CC = 0.36, *p* = 0.04) protein detection—a protein marker linked to cellular stress response, particularly oxidative stress. Interestingly, *Porphyromonas* is positively correlated with all three clinical measurements, with the highest correlation observed with MGI (Spearman CC = 0.48, *p* < 0.001), a measurement of gingival inflammation (Spearman CC = 0.60, *p* = 0.006). It was also positively correlated with CD55 (Spearman CC = 0.38, *p* = 0.03) and *GSR* (Spearman CC = 0.36, *p* = 0.04) levels and negatively correlated with KLK12 (Spearman CC = −0.37, *p* = 0.04). *Rothia* did not show a significant correlation with clinical measurements; however, it did exhibit a significant negative correlation with *Porphyromonas* (Spearman CC = −0.40, *p* = 0.005) and *Fusobacterium* (Spearman CC = −0.57, *p* = 0.02). The proteins significantly correlated with clinical measurements were positively linked. These proteins span the areas of stress response, keratinocyte differentiation, bacterial infection, oxidative response, and inflammation (Figure 2), as well as disease association (Figure 3). High expression of these proteins is associated with elevated clinical measurements of GBI, BLD, and MGI.

### 3.5. Treatment Effect on the Supragingival Plaque Microbiome in High Bleeders

**Figure 5:**
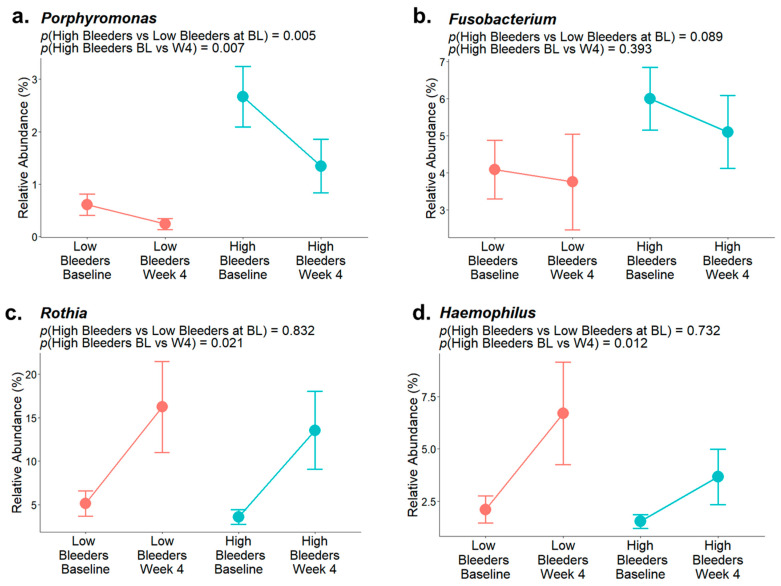
Representative bacterial genera relative abundance of low and high bleeders at baseline and after four weeks of SnF_2_ toothpaste treatment. (**a**) Relative abundance of *Porphyromonas.* (**b**) Relative abundance of *Fusobacterium*. (**c**) Relative abundance of *Rothia.* (**d**) Relative abundance of *Haemophilus*.

After a four-week use of SnF_2_-containing toothpaste, 17 bacterial genera were found to be differently abundant (Table S2) compared with baseline. In particular, the relative abundance of *Porphyromonas* (Figure 5a) significantly decreased, *Fusobacterium* (Figure 5b) decreased, and *Rothia* (Figure 5c) and *Haemophilus* (Figure 5d) significantly increased. These changes/shifts in the supragingival microbiome composition after SnF_2_ use are consistent with improved clinical signs and coincide with decreased bleeding and inflammation indices reported previously [33]. In conclusion, SnF_2_ treatment positively influenced the supragingival microbiomes of individuals with unhealthy or heavy bleeding gums.

### 3.6. Impact of SnF_2_ Toothpaste After Eight Weeks of Treatment in High Bleeders

**Figure 6:**
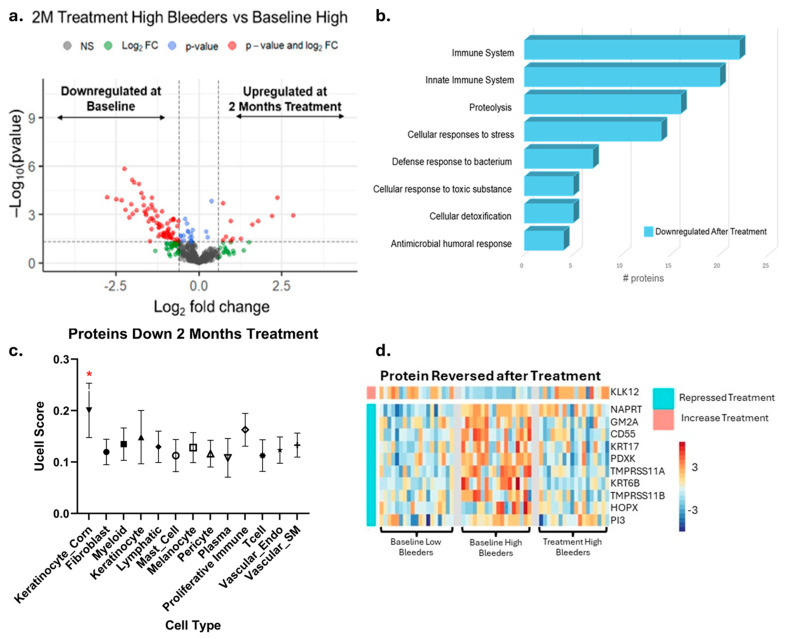
Proteomics and single-cell integration analyses reveal the impact of eight weeks’ use of SnF_2_ toothpaste in high bleeders. (**a**) The volcano plot displays differentially expressed proteins in high bleeders after treatment compared with baseline, using a fold change (FC) ≥ 1.5 and a *p*-value ≤ 0.05 as criteria. The x axis represents a log2 fold change. The y axis represents −log10 (*p* value). Grey dots represent proteins without significant changes (NS: not significant); red dots indicate proteins with a significant fold change (*p* ≤ 0.05 and FC ≥ 1.5); blue dots represent proteins with significant changes (*p* ≤ 0.05) but with FC < 1.5; green dots signify proteins with FC ≥ 1.5 but not significant (*p* > 0.05). (**b**) Enrichment analysis of the differentially expressed proteins highlights the biological processes associated with proteins that are more enriched in high bleeders compared with low bleeders. (**c**) UCELL score of proteins downregulated after eight weeks of treatment. * Two-way ANOVA followed by Kruskal-Wallis test *q* < 0.01 (**d**) Specifically, ten proteins enriched in cornifying keratinocytes, initially increased in high bleeders, were downregulated after treatment.

The proteomics data revealed a total of 80 proteins that were significantly different in high bleeders when comparing eight weeks of treatment to baseline. Of these, 69 proteins were downregulated after treatment, while 11 were upregulated (Figure 6a, Supplementary Tables S6 and S7). An enrichment analysis revealed that the downregulated proteins are primarily involved in proteolysis, antimicrobial humoral responses, cellular responses to stress, and immune system processes (Figure 6b).

Proteins with established antimicrobial functions, such as CD55, HTN3, CHIT1, PI3, and GBP6, were upregulated at baseline in high bleeders, but their levels decreased after eight weeks of treatment. This pattern suggests that high bleeders initially have higher levels of these antimicrobial proteins as part of their body’s defense response. The subsequent reduction in protein levels following treatment may indicate that the treatment is effective in reducing inflammation and/or bacterial load.

The gene signatures of the proteins suppressed after stannous treatment were again overlaid onto single-cell data and revealed the cornifying keratinocytes (keratinocyte_corn) cluster as being enriched (Figure S2). Specifically, ten proteins initially increased in high bleeders and then reversed after treatment were enriched in cornifying keratinocytes and included the barrier disruption indicators KRT6B and KRT17 (Figure 6d). Together, the corresponding genes of 15 proteins identified as being repressed by treatment were selectively expressed in cornifying keratinocytes. Repression and/or reversal of these proteins indicates that the initial epidermal barrier disruption and likely corresponding bacterial infection is alleviated after treatment.

Notably, many of these downregulated proteins are associated with immune and inflammatory responses, including several immunoglobulin chains (such as IGKV6D-21, IGHV3-35, and IGLV3-21) and antimicrobial proteins (such as S100A8, S100A9, and CD55). The reduction in these proteins suggests that the treatment of gingivitis may attenuate local immune activation and inflammatory processes.

In addition to immune-related proteins, several enzymes and regulatory proteins involved in proteolysis and cellular stress responses were also downregulated after treatment. For example, proteasome subunits (PSMA3, PSMB1, PSME2), heat shock proteins (HSP90AA1, HSP90B1, HSPA4), and metabolic enzymes (AKR1B10, ALDH1A1, IDH1) all showed decreased expression. This pattern may reflect a broader suppression of protein turnover, stress signaling, and metabolic activity in response to the treatment. Such changes indicate a shift toward a more balanced oral environment, which may promote healing and overall oral health.

Decreased levels of SERPINB2 and S100 family proteins (S100A8, S100A9, S100A12) point to a marked attenuation of the inflammatory response. In parallel, the downregulation of proteins involved in cellular protection against oxidative stress, such as TXNDC17 and APEX1, suggests that the treatment is also effective in reducing oxidative damage. Together, these changes indicate that oral intervention may modulate immune activity and may also contribute to a less inflammatory and less oxidative oral environment, potentially supporting improved tissue health and resilience.

### 3.7. Treatment Reduced Collagen Degradation, Consistent with the Inhibitory Effects of SnF_2_ on Bacterial Collagenases

**Figure 7:**
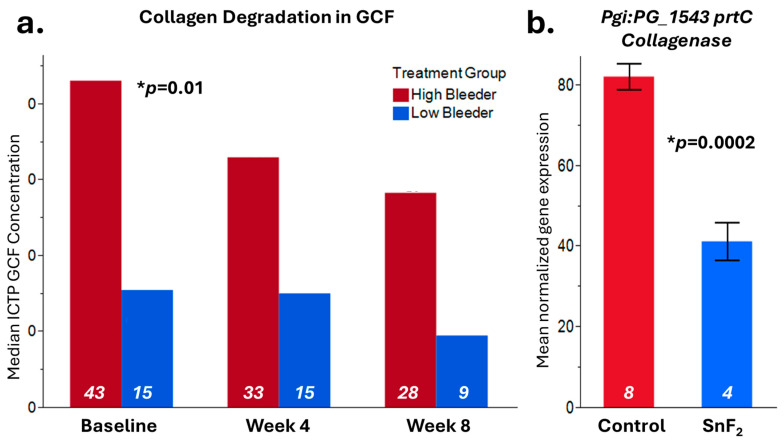
Collagen degradation results. (**a**) Crosslinked carboxyterminal telopeptide of type I collagen (ICTP) from GCF (gingival crevicular fluid) samples was measured at baseline, Week 4, and Week 8 in both high bleeders and low bleeders. The median value is displayed in the graph, significant differences at baseline between high and low bleeder samples were observed with *p* = 0.01. (**b**) In vitro, the RNASeq result [34] showed that the *P. gingivalis* collagenase *prtC* gene expression is inhibited by SnF_2_ treatment with *p* = 0.0002. Sample numbers for each leg are listed inside the bar with white color.

In this research, we set out to test CTP, as a measure of collagen breakdown in the mouth. The ICTP value at baseline was significantly higher in high bleeders (gingivitis group) vs. low bleeders (healthy group) in GCF (*p* = 0.01) (Figure 7a). A significant reduction in GCF ICTP over baseline was observed at eight weeks after the use of SnF_2_ dentifrice in both high (*p* ≤ 0.001) and low bleeders (*p* ≤ 0.001). A significant positive correlation between GCF ICTP and bleeding (r = 0.51, *p* = 0.0013), as well as the modified gingival index (r = 0.50, *p* = 0.0021), was observed. We also observed a significant positive correlation of ICTP with markers that were measured earlier [33], namely lactate dehydrogenase (LDH) (0.52, *p* < 0.0001), IL-6 (0.25, *p* = 0.01), and oxidized LDL (oxLDL) (0.37, *p* = 0.016)—which is a systemic oxidative stress marker in saliva—which are risk factors of CVD.

In our previous publication, we reported that the *F. nucleatum* Collagenase *prtC* gene was significantly inhibited by SnF_2_ treatment [34] (Figure 7b). We also observed that *P. gingivalis prtC* gene expression was significantly downregulated by 0.0908% SnF_2_ treatment (*p* = 0.0002). Consistent with the GCF collagen degradation measurement, both results indicated that SnF_2_ reduces collagen degradation potentially by inhibiting pathogen collagenase activities or reducing bacterial collagenase production.

## 4. Discussion

### 4.1. Therapeutic Effects of SnF_2_ Dentifrice

Collectively, our results suggest that SnF_2_ dentifrice treatment induced favorable changes in the microbial composition in high bleeders by reducing the relative abundance of pathogenic bacteria, such as *Porphyromonas* and *Fusobacterium*, which are typically higher in individuals with poor oral health. Commensal bacterial genera, such as *Rothia* and *Haemophilus*, showed an increased relative abundance following SnF_2_ treatment (Figure 1 and Figure 5). Although *Haemophilus* encompasses both commensal and pathogenic species, recent literature identified *Haemophilus parainfluenzae* as highly abundant and active commensal in the healthy oral cavity [41]. These shifts to a healthier microbiome could lead to reduced inflammation and oxidative stress. The proteomic analyses highlight significant downregulation of pro-inflammatory cytokines and enzymes, such as IL-36G and MMPs, which are known to exacerbate tissue inflammation and degradation. The presence of increased oxidative stress markers at baseline in high bleeders, such as SOD2 and GSR, indicates a compensatory response to elevated ROS levels, which can lead to cellular damage and exacerbate inflammatory processes. The reduction of oxidative stress markers, as evidenced by the downregulation of antioxidant enzymes, such as SOD2 and GSR, supports the hypothesis that SnF_2_ can effectively mitigate the inflammatory state associated with gingivitis and reduce oxidative stress (Figure 2 and Figure 4). A compromised epithelial barrier observed in high bleeders is indicated by the upregulation of keratinocyte markers associated with barrier disruption (Figure 2). When these barriers are impaired, inflammatory mediators and bacterial toxins can translocate into the bloodstream, exacerbating systemic inflammation and contributing to the pathogenesis of various diseases [42,43,44,45]. The proteins CD55, GBP6, IGHV3-35, and IGKV1-6 (Supplementary Tables S3 and S6) are significant markers in the context of the host response to bacterial infections, particularly in relation to gingivitis and treatment outcomes. Their elevated levels in high bleeders compared with low bleeders at baseline suggest a strong association with the inflammatory response characteristic of gingivitis. Notably, the reduction of these proteins in high bleeders after eight weeks of SnF_2_ therapy indicates a potential therapeutic effect, highlighting their role in modulating inflammation and the immune response. CD55, known as decay-accelerating factor, is crucial for regulating both innate and adaptive immunity by preventing excessive complement activation and promoting the resolution of inflammation [46]. GBP6 serves as a central regulator of inflammation and immune response to infections [47], while IGHV3-35 and IGKV1-6 are vital for the specificity of immunoglobulins against bacterial antigens [48]. Collectively, these findings underscore the mechanistic involvement of these proteins in the host’s response to bacterial infection during gingivitis, suggesting their potential as biomarkers for disease severity and treatment efficacy. The integration of functional assays with single-cell transcriptomic data reveals that SnF_2_ treatment enhances the barrier properties of keratinocytes, as evidenced by the downregulation of markers associated with barrier disruption (e.g., KRT6B and KRT17). Such improvements in epithelial integrity are vital, as they prevent pathogenic infiltration and promote effective healing responses in the oral mucosa (Figure 3 and Figure 5).

Collagen has a significant impact on the overall health of the body and is an essential protein in the human body, offering multiple health benefits and functions [49]. Increased collagen breakdown can lead to sagging skin and, in extreme cases, even tooth loss. Collagen degradation is closely linked to connective tissue weakness and accelerated biological aging. Three types of collagens account for 90% of the collagen genetic makeup: type I, type II, and type III [50]. Collagen repair and regeneration is a continual process. With chronic inflammation, however, collagen and tissue repair are compromised. In the mouth, collagen type I is found within gingival tissue, arranged in either tightly parallel patterns or disorganized structures, with the presence of inflammatory cells in the case of gingivitis [51]. As collagen begins to break down, smaller peptides become available. These peptides include amino N- and carboxy C-terminal chains. As alluded to earlier, gingivitis leads to an increase in gram-negative bacteria at the surface of the gum line, and lipopolysaccharides (LPSs) or endotoxins permeate into the soft tissue. This triggers the body’s immune response, where polymorphonuclear leukocytes (PMNs) are recruited to the site of infection. Part of the functional pathways of PMNs is the production of collagen degradation enzymes and MMPs. In parallel, LPS triggers the secretion of many cytokines, including interleukin-1 (IL-1) and tumor necrosis factor (TNF), which also trigger further tissue destruction. As the destruction of tissues progresses, the release of peptides, like ICTP, continues to concentrate and be expelled via the GCF and further diluted in saliva. As collagen breaks down, it has the potential to promote the release of more bacterial toxins into the bloodstream, thereby enhancing systemic inflammation.

**Figure 8:**
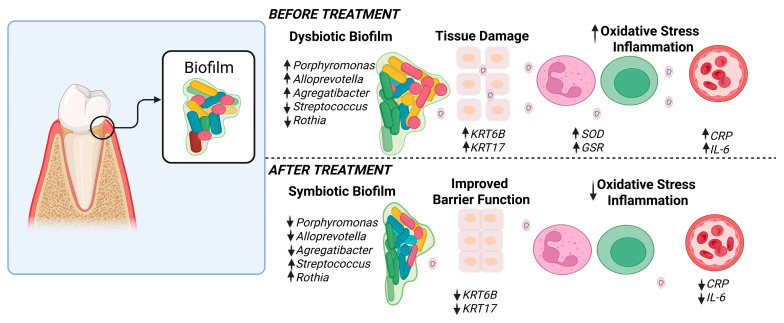
Hypothesis of host microbial changes. This schematic illustration depicts the hypothesized alterations before and after SnF_2_ treatment. Before treatment, there is an increased biofilm accumulation associated with microbiota dysbiosis and a higher prevalence of pathogenic species in the high-bleeder sites. Additionally, dominant protein changes are linked to barrier/tissue damage, oxidative stress, and inflammation. After treatment, the biofilm composition changed. It is hypothesized that biofilm transitions to a symbiotic state, resulting in improved barrier function and reduced oxidative stress and inflammation. Figure created in BioRender.com.

Collagen is crucial for maintaining the structural integrity of oral tissues, and its breakdown can lead to compromised connective tissue health and accelerated aging. Our findings indicate that SnF_2_ treatment significantly reduces the levels of crosslinked carboxyterminal telopeptide of type I collagen (ICTP), a marker of collagen degradation in gingival crevicular fluid (GCF). This reduction is indicative of the ability of SnF_2_ to preserve tissue integrity by inhibiting the microbial degradative enzymes. These microbial and proteomic changes correlated strongly with the clinical measurements, showing significant reductions in bleeding and inflammation indices (Figure 6 and Figure 7) following eight weeks of SnF_2_ treatment. Furthermore, the overlap between salivary protein biomarkers and disease markers suggests that these consistent biomarkers may represent a group of systemic health indicators, or potential shared mechanisms underlying inflammation throughout the body. The findings from this study indicate that the proteomic markers associated with gingivitis in high bleeders significantly overlap with numerous plasma protein markers elevated in various diseases, as identified in the UK Biobank study. Specifically, 71 of the baseline gingivitis proteomic biomarkers correlated with those linked to conditions such as infectious diseases, cardiovascular issues, diabetes, dementia, and depression. Notably, after 2 months of SnF_2_ treatment, there was a significant reduction in 29 of these biomarkers among high bleeders, with 25–40% of the overlapping disease biomarkers demonstrating a shift towards a healthier status (Figure 3). There is support in the literature for systematic inflammation that can be triggered by infection, trauma, surgery, chronic disease, or even psychological stress [52,53,54,55]. The core mechanism involves the release of proinflammatory cytokines and activation of immune pathways that affect multiple organs and tissues throughout the body. Collectively, microbiome dysbiosis can lead to a cascade of inflammatory responses that extend beyond the oral cavity. The presence of pathogenic bacteria, as observed in high bleeders, contributes to the secretion of inflammatory proteins and cytokines, which can enter the systemic circulation [56]. This not only exacerbates local inflammation but also activates systemic signaling pathways that may contribute to the development and progression of conditions such as cardiovascular disease, diabetes, and other inflammatory disorders. Vice versa, systematic disease can also impact the oral microbiome and increase susceptibility to oral disease [57]. Our findings indicate that SnF_2_ treatment modifies oral microflora by decreasing pathogenic bacteria and increasing beneficial commensals, which in turn reduces tissue damage and oxidative stress (Figure 8). This leads to the repair of the epithelial barrier, thereby preventing the penetration of bacteria and toxins into the bloodstream. Maintaining the integrity of this barrier is crucial, as it offers the desirable benefit of keeping harmful bacteria and toxins out of the circulation, ultimately helping to reduce the potential for systemic damage.

### 4.2. Novelty and Advantages of the Multi-Omics Approach

The application of a multi-omics approach in this study has proven invaluable in providing a comprehensive understanding of the intricate interactions between oral microbiota, proteomic changes, and systemic health. Integrating proteomics and single-cell data associated with plasma disease markers has enabled us to elucidate new connections, revealing how changes at the cellular level in the oral environment can propagate systemic effects. This integrative strategy not only enhances our understanding of how dysbiosis influences inflammatory responses and systemic health outcomes but also enables the identification of specific biomarkers that can serve as targets for therapeutic interventions.

### 4.3. Limitations and Considerations, Future Direction, and Impact

While this study provides significant insights into the relationship between oral health and systemic conditions through a multi-omics approach, it is important to acknowledge certain limitations. The observed connections between single-cell data, proteomic changes, and systemic diseases do not establish direct causative effects. Further research is essential to validate these findings and explore the additional dimensions of the oral–systemic health connection, including longitudinal studies that assess the long-term effects of interventions like SnF_2_ on both oral and systemic health. Moreover, the relationship between oral and systemic diseases is complex and multiple contributing factors exist, including genetics, environmental factors, lifestyle, stress, etc. As we discussed earlier, oral diseases can influence systemic health, while systemic diseases can also worsen oral health by altering immune response, inflammation, and the oral microbiome. This connection is increasingly recognized as bidirectional. Investigating specific patient populations that are at risk for systemic diseases could also yield valuable insights into tailored treatment strategies. Our results also suggest that oral changes serve as indicators of systemic health, providing opportunities for the development of detection and alerting methods to assist both patients and healthcare professionals in identifying systemic diseases earlier, thereby facilitating preventive care. This knowledge can facilitate a more integrated approach to patient care, emphasizing the need for collaboration among dental and medical professionals to address the broader implications of oral health on overall well-being. Additionally, this type of study could inform the development of targeted therapeutic agents with improved biocompatibility and minimal side effects [58] and explore specific compounds that may address systemic conditions related to oral health [59].

## 5. Conclusions

SnF_2_ dentifrice treatment positively impacts the oral microbiome by reducing pathogenic bacteria, like *Porphyromonas* and *Fusobacterium*, while increasing beneficial commensals, such as *Rothia* and *Haemophilus*, in high bleeders. These changes are associated with decreased inflammation, oxidative stress, and tissue damage, as indicated by the downregulation of pro-inflammatory cytokines and biomarkers. SnF_2_ also enhances epithelial barrier integrity. This study highlights the relationship between oral health and systemic conditions, indicating that enhancing gingival health through SnF_2_ may help to support a healthier balance of oral microbiota, decrease harmful microbial effects, and alleviate inflammation, potentially benefiting overall health. The multi-omics approach employed here suggests possible biomarkers for therapeutic interventions and points to the value of considering integrated dental and medical care.

## Data Availability

The 16S data has been deposited into the NCBI SRA (BioProject ID PRJNA1345442). The remaining datasets are not publicly available as they are considered proprietary, but may be available from the corresponding author on reasonable request.

## Supplementary Materials

The following supporting information can be downloaded at: https://www.mdpi.com/article/10.3390/microorganisms13102371/s1. Table S1. Baseline demographic and clinical characteristics reported by Ramji et al. [33]*. Table S2. Plaque Microbial Composition and Statistical Analysis Result. Table S3. Proteins Showed Significant Differences between High Bleeders vs. Low Bleeders at Baseline. Table S4. GO Biological Process Showed Significant Differences between High Bleeders vs. Low Bleeders at Baseline. Table S5. Example Proteins with Significant Differences in this Study and Their Disease Association from “Atlas of the plasma proteome” [27]. Table S6. Proteins Showed Significant Differences after Eight Weeks Treatment in High Bleeders (2 Months vs. Baseline). Table S7. GO Biological Process Showed Significant Differences After 8 Weeks Treatment in High Bleeders. Figure S1. Single cell overlay of baseline Protein changes. (a) Single cell data from control buccal and gingival biopsies, previously published, was downloaded, reprocessed and annotated. (b) Ucell analyses of Proteins up or down regulated in Salivary samples are enriched in Immune cells and Cornifying Keratinocytes. (c,d) After subsetting the major Immune clusters into more specific cell populations, it was revealed that specifically proteins from Neutrophils and mDC cells were specifically enriched in saliva of high bleeders. Figure S2. Single cell data from control buccal and gingival biopsies were used to predict major cell types affected by SnF_2_ treatment. Ucell analyses of proteins downregulated after eight weeks of treatment show their corresponding gene expression was enriched in Cornifying Keratinocytes.

## References

[1] World Health Organization Global Oral Health Status Report: Towards Universal Health Coverage for Oral Health by 2030. https://www.who.int/publications/i/item/9789240061484.

[2] Radaic A., Kapila Y.L. (2021). The oralome and its dysbiosis: New insights into oral microbiome-host interactions. Comput. Struct. Biotechnol. J..

[3] Socransky S.S., Haffajee A.D., Cugini M.A., Smith C., Kent R.L. (1998). Microbial complexes in Supragingival plaque. J. Clin. Periodontol..

[4] Socransky S.S., Haffajee A.D. (2005). Periodontal microbial ecology. Periodontology 2000.

[5] Darveau R. (2010). Periodontitis: A polymicrobial disruption of host homeostasis. Nat. Rev. Microbiol..

[6] Hajishengallis G., Lamont R.J. (2012). Beyond the red complex and into more complexity: The polymicrobial synergy and dysbiosis (PSD) model of periodontal disease etiology. Mol. Oral Microbiol..

[7] Holt S.C., Ebersole J.L. (2005). Porphyromonas Gingivalis, Treponema Denticola, and Tannerella Forsythia: The ‘Red Complex’, a prototype polybacterial pathogenic consortium in periodontitis. Periodontology 2000.

[8] Sedghi L.M., Bacino M., Kapila Y.L. (2021). Periodontal disease: The good, the bad, and the unknown. Front. Cell. Infect. Microbiol..

[9] Gasmi Benahmed A., Kumar Mujawdiya P., Noor S., Gasmi A. (2022). Porphyromonas Gingivalis in the development of periodontitis: Impact on dysbiosis and inflammation. Arch. Razi. Inst..

[10] Brennan C.A., Garrett W.S. (2019). Fusobacterium nucleatum—Symbiont, opportunist and oncobacterium. Nat. Rev. Microbiol..

[11] Afzoon S., Amiri M.A., Mohebbi M., Hamedani S., Farshidfar N. (2023). A systematic review of the impact of Porphyromonas gingivalis on foam cell formation: Implications for the role of periodontitis in atherosclerosis. BMC Oral Health.

[12] Seyedmoalemi M.A., Saied-Moallemi Z. (2025). Association between periodontitis and Alzheimer’s disease: A narrative review. IBRO Neurosci. Rep..

[13] Bui F.Q., Almeida-da-Silva C.L.C., Huynh B., Trinh A., Liu J., Woodward J., Asadi H., Ojcius D.M. (2019). Association between periodontal pathogens and systemic disease. Biomed. J..

[14] Li L., Zhang Y.-L., Liu X.-Y., Meng X., Zhao R.-Q., Ou L.-L., Li B.-Z., Xing T. (2021). Periodontitis exacerbates and promotes the progression of chronic kidney disease through oral flora, cytokines, and oxidative stress. Front. Micro.

[15] Li X., Kolltveit K.M., Tronstad L., Olsen I. (2000). Systemic diseases caused by oral infection. Clin. Microbiol. Rev..

[16] Iwashita M. (2023). Association between periodontal disease and arteriosclerosis-related diseases. J. Atheroscler. Thromb..

[17] Li X., Kiprowska M., Kansara T., Kansara P., Li P. (2022). Neuroinflammation: A distal consequence of periodontitis. J. Dent. Res..

[18] Liu S., Butler C.A., Ayton S., Reynolds E.C., Dashper S.G. (2024). *Porphyromonas gingivalis* and the pathogenesis of Alzheimer’s disease. Crit. Rev. Microbiol..

[19] Haditsch U., Roth T., Rodriguez L., Hancock S., Cecere T., Nguyen M., Arastu-Kapur S., Broce S., Raha D., Lynch C.C. (2020). Alzheimer’s Disease-Like neurodegeneration in Porphyromonas gingivalis infected neurons with persistent expression of active gingipains. J. Alzheimers Dis..

[20] Chen C., Wang J., Pan D., Wang X., Xu Y., Yan J., Wang L., Yang X., Yang M., Liu G.P. (2023). Applications of multi-omics analysis in human diseases. MedComm.

[21] Huang S., He T., Yue F., Xu X., Wang L., Zhu P., Teng F., Sun Z., Liu X., Jing G. (2021). Longitudinal multi-omics and microbiome meta-analysis identify an asymptomatic gingival state that links gingivitis, periodontitis, and aging. mBio.

[22] Xu P., Gunsolley J. (2014). Application of metagenomics in understanding oral health and disease. Virulence.

[23] Klukowska M., Ramji N., Muñoz Bodnar A., Hu P., Ye H., Xie S., Li L., Ashe J., Reichling T., Wang J. (2025). Clinical effects of stannous fluoride dentifrice on peri-implant mucositis, plaque microbiome, and oxidative stress. Am. J. Dent..

[24] Belstrøm D., Jersie-Christensen R.R., Lyon D., Damgaard C., Jensen L.J., Holmstrup P., Olsen J.V. (2016). Metaproteomics of saliva identifies human protein markers specific for individuals with periodontitis and dental caries compared to orally healthy controls. PeerJ.

[25] Bao K., Li X., Poveda L., Qi W., Selevsek N., Gumus P., Emingil G., Grossmann J., Diaz P.I., Hajishengallis G. (2020). Proteome and microbiome mapping of human gingival tissue in health and disease. Front. Cell. Infect. Microbiol..

[26] Balachandran M., Cross K.L., Podar M. (2020). Single-Cell genomics and the oral microbiome. J. Dent. Res..

[27] Deng Y.T., You J., He Y., Zhang Y., Li H.Y., Wu X.R., Cheng J.Y., Guo Y., Long Z.W., Chen Y.L. (2025). Atlas of the plasma proteome in health and disease in 53,026 adults. Cell.

[28] Nguyen D.H., Chu D.T., Mani I., Singh V. (2024). Multi-omics in Study of Oral Microbiome. Multi-Omics Analysis of the Human Microbiome.

[29] Biesbrock A., He T., DiGennaro J., Zou Y., Ramsey D., Garcia-Godoy F. (2019). The effects of bioavailable gluconate chelated stannous fluoride dentifrice on gingival bleeding: Meta-analysis of eighteen randomized controlled trials. J. Clin. Periodontol..

[30] Chen D., Chew D., Xiang Q., Lam T., Dai Y., Liu J., Wang L., He T., Strand R., Zhang X. (2024). Interactions and effects of a stannous-containing sodium fluoride dentifrice on oral pathogens and the oral microbiome. Front. Microbiol..

[31] Xie S., Tansky C.S., Ashe J., Gao F., Ramji N.B., Iberi V., Sun Y., Ramji N., Biesbrock A.R. (2024). Stannous fluoride protects gingival keratinocytes against infection and oxidative stress by *Porphyromonas gingivalis* outer membrane vesicles. Front. Dent. Med..

[32] Haught C., Xie S., Circello B., Tansky C.S., Khambe D., Klukowska M., Huggins T., White D.J. (2016). Lipopolysaccharide and lipoteichoic acid virulence deactivation by stannous fluoride. J. Clin. Dent..

[33] Ramji N., Xie S., Bunger A., Trenner R., Ye H., Farmer T., Reichling T., Ashe J., Milleman K., Milleman J. (2024). Effects of stannous fluoride dentifrice on gingival health and oxidative stress markers: A prospective clinical trial. BMC Oral Health.

[34] Hu P., Xie S., Shi B., Tansky C.S., Circello B., Sagel P.A., Schneiderman E., Biesbrock A.R. (2024). The effect of oral care product ingredients on oral pathogenic bacteria transcriptomics through RNA-Seq. Microorganisms.

[35] Chalita M., Kim Y.O., Park S., Oh H.S., Cho J.H., Moon J., Baek N., Moon C., Lee K., Yang J. (2024). EzBioCloud: A genome-driven database and platform for microbiome identification and discovery. Int. J. Syst. Evol. Microbiol..

[36] Williams D.W., Greenwell-Wild T., Brenchley L., Dutzan N., Overmiller A., Sawaya A.P., Webb S., Martin D., Hajishengallis G., NIDCD/NIDCR Genomics and Computational Biology Core (2021). Human oral mucosa cell atlas reveals a stromal-neutrophil axis regulating tissue immunity. Cell.

[37] Hao Y., Hao S., Andersen-Nissen E., Mauck W.M., Zheng S., Butler A., Lee M.J., Wilk A.J., Darby C., Zager M. (2021). Integrated analysis of multimodal single-cell data. Cell.

[38] Andreatta M., Carmona S.J. (2021). UCell: Robust and scalable single-cell gene signature scoring. Comput. Struct. Biotechnol. J..

[39] Chen J., Bardes E.E., Aronow B.J., Jegga A.G. (2009). ToppGene Suite for gene list enrichment analysis and candidate gene prioritization. Nucleic Acids Res..

[40] Zhang X., Yin M., Zhang L. (2019). Keratin 6, 16 and 17—Critical barrier alarmin molecules in skin wounds and psoriasis. Cells.

[41] Giacomini J.J., Torres-Morales J., Tang J., Dewhirst F.E., Borisy G.G., Mark Welch J.L. (2024). Spatial ecology of Haemophilus and Aggregatibacter in the human oral cavity. Microbiol. Spectr..

[42] Groeger S.E., Meyle J. (2015). Epithelial barrier and oral bacterial infection. Periodontology 2000.

[43] Förster C. (2008). Tight junctions and the modulation of barrier function in disease. Histochem. Cell Biol..

[44] Galea I. (2021). The blood-brain barrier in systemic infection and inflammation. Cell. Mol. Immunol..

[45] Merga Y., Campbell B.J., Rhodes J.M. (2014). Mucosal barrier, bacteria and inflammatory bowel disease: Possibilities for therapy. Dig. Dis..

[46] Dho S.H., Lim J.C., Kim L.K. (2018). Beyond the Role of CD55 as a Complement Component. Immune Netw..

[47] Tretina K., Park E.S., Maminska A., MacMicking J.D. (2019). Interferon-induced guanylate-binding proteins: Guardians of host defense in health and disease. J. Exp. Med..

[48] Hatzi K., Catera R., Moreno Atanasio C., Fischetti V.A., Allen S.L., Kolitz J.E., Rai K.R., Chu C.C., Chiorazzi N. (2016). Chronic lymphocytic leukemia immunoglobulins display bacterial reactivity that converges and diverges from auto-/poly-reactivity and IGHV mutation status. Clin. Immunol..

[49] Pillai N.S., Khan A.K., Mehrotra N., Jadhav K. (2024). A comprehensive review on the role of collagen in health and disease. Biotech. Res. Asia.

[50] Chacon E.L., Bertolo M.R.V., de Guzzi Plepis A.M., da Conceição Amaro Martins V., Dos Santos G.R., Pinto C.A.L., Pelegrine A.A., Teixeira M.L., Buchaim D.V., Nazari F.M. (2023). Collagen-chitosan-hydroxyapatite composite scaffolds for bone repair in ovariectomized rats. Sci. Rep..

[51] Almeida T., Valverde T., Martins-Júnior P., Ribeiro H., Kitten G., Carvalhaes L. (2015). Morphological and quantitative study of collagen fibers in healthy and diseased human gingival tissues. Rom. J. Morphol. Embryol..

[52] Walker K.A., Ficek B.N., Westbrook R. (2019). Understanding the role of systemic inflammation in Alzheimer’s Disease. ACS Chem. Neurosci..

[53] Margraf A., Ludwig N., Zarbock A., Rossaint J. (2020). Systemic inflammatory response syndrome after surgery: Mechanisms and protection. Anesth. Analg..

[54] Xia Y., Xia C., Wu L., Li Z., Li H., Zhang J. (2023). Systemic Immune Inflammation Index (SII), System Inflammation Response Index (SIRI) and risk of all-cause mortality and cardiovascular mortality: A 20-year follow-up cohort study of 42,875 US adults. J. Clin. Med..

[55] Kocamer Şahin Ş., Aslan E. (2024). Inflammation as a neurobiological mechanism of cognitive impairment in psychological stress. J. Integr. Neurosci..

[56] Kleinstein S.E., Nelson K.E., Freire M. (2020). Inflammatory networks linking oral microbiome with systemic health and disease. J. Dent. Res..

[57] Graves D.T., Corrêa J.D., Silva T.A. (2019). The Oral Microbiota Is Modified by Systemic Diseases. J. Dent. Res..

[58] Chiriac A.P., Diaconu A., Nita L.E., Tudorachi N., Mititelu-Tartau L., Creteanu A., Dragostin O., Rusu D., Popa G. (2017). The influence of excipients on physical and pharmaceutical properties of oral lyophilisates containing a pregabalin-acetaminophen combination. Expert Opin. Drug Deliv..

[59] Anton I.C., Mititelu-Tartau L., Popa E.G., Poroch M., Poroch V., Pelin A.M., Pavel L.L., Drochioi I.C., Botnariu G.E. (2022). Zinc Chloride Enhances the Antioxidant Status, Improving the Functional and Structural Organic Disturbances in Streptozotocin-Induced Diabetes in Rats. Medicina.

