# Supplementary material for: Multi-Omics Insights into Gingivitis from a Clinical Trial: Understanding the Role of Bacterial and Host Factors
Source: Microorganisms. 2025 Oct 15;13(10):2371. doi: 10.3390/microorganisms13102371 (PMC12566121; doi:10.3390/microorganisms13102371)
Supplement: Supplementary file 1 [file microorganisms-13-02371-s001.zip › Supplementary Tables.pdf]

microorganisms MDPI

Supplementary Tables:

Table S1. Baseline demographic and clinical characteristics reported by Ramji et al. [33]*

Demographic/Statistic or Category	Healthy ($n = 19$)	Disease ($n = 20$)	Overall ($n = 39$)	p-value
**Age (Years)**				
Mean (SD)	51.00 (14.674)	52.95 (11.473)	52.00 (12.992)	0.6456[a]
Min.-Max	28—73	25—67	25—73	
**Ethnicity**				
Not Hispanic or Latino[b]	19 (100%)	20 (100%)	39 (100%)	
**Race**				
American Indian or Alaskan Native[b]	1 (5%)	1 (5%)	2 (5%)	0.4658[c]
Asian[b]	0 (0%)	1 (5%)	1 (3%)	
Black or African American[b]	2 (11%)	0 (0%)	2 (5%)	
Multiracial[b]	1 (5%)	0 (0%)	1 (3%)	
White/Caucasian[b]	15 (79%)	18 (90%)	33 (85%)	
**Sex**				
Female[b]	17 (89%)	17 (85%)	34 (87%)	1.0000[c]
Male[b]	2 (11%)	3 (15%)	5 (13%)	
**Smoking status**				
Yes[b]	1 (5%)	1 (5%)	2 (5%)	1.0000[c]
No[b]	18 (95%)	19 (95%)	37 (95%)	
**Clinical Characteristics**				
Bleeding sites, mean (SD)	2.00 (1.202)	33.10 (9.624)	17.95 (17.175)	< 0.001[d]
MGI score, mean (SD)	1.28 (0.320)	2.48 (0.359)	1.89 (0.692)	< 0.001[d]

*Reproduced per Creative Commons license http://creativecommons.org/licenses/by/4.0/.
[a]Two-sided ANOVA p-value for the group comparison
[b]The number (percent) of participants in each category
[c]Two-sided chi-square p-value for the group comparison
[d]Two-sided Fisher's exact test p-value for the group comparison

Table S2. Plaque Microbial Composition and Statistical Analysis Result

**Genus**	**Average Relative Abundance**			**Fold Change**		**Wilcoxon *P* value** unpaired	**Wilcoxon *P* value** paired	**FDR Adjusted *p*-value**		**Baseline Spearman Correlation *p* value**		
	**Low BL**	**High BL**	**W4 High**	**High BL vs Low BL**	**High W4 vs High BL**	**High BL vs Low BL**	**High W4 vs High BL**	**High BL vs Low BL**	**High W4 vs High BL**	**GBI**	**BLD**	**MGI**
Neisseriaceae_uc	0.01	0.20	0.09	21.13	-2.33	0.00	0.20	0.11	0.67	0.00	0.00	0.00
Porphyromonas	0.61	2.67	1.34	4.38	-1.99	0.00	0.01	0.11	0.14	0.00	0.01	0.00
Ottowia	0.01	0.13	0.37	9.32	2.80	0.02	0.45	0.20	0.75	0.07	0.04	0.04
Alloprevotella	0.24	0.36	0.67	1.50	1.85	0.02	0.67	0.20	0.89	0.06	0.02	0.03
Megasphaera	0.05	0.20	0.17	3.76	-1.18	0.02	0.81	0.20	0.93	0.04	0.06	0.03
Moryella	0.07	0.26	0.09	3.64	-2.79	0.02	0.02	0.20	0.16	0.04	0.04	0.10
Granulicatella	0.67	1.19	0.85	1.77	-1.40	0.04	0.06	0.26	0.38	0.03	0.04	0.02
Propionibacterium	0.00	0.57	0.46	709.78	-1.25	0.05	0.44	0.27	0.75	0.03	0.02	0.08
Olsenella	0.05	0.14	0.10	3.16	-1.47	0.09	0.63	0.37	0.89	0.05	0.04	0.13
Fusobacterium	4.09	6.00	5.11	1.47	-1.18	0.09	0.39	0.37	0.72	0.07	0.10	0.04
Treponema	0.10	0.18	0.32	1.82	1.76	0.10	0.27	0.37	0.69	0.04	0.04	0.14
Leptotrichia	7.43	6.46	2.90	-1.15	-2.23	0.71	0.00	0.83	0.14	0.38	0.35	0.92
Abiotrophia	0.11	0.24	0.09	2.31	-2.59	0.47	0.01	0.70	0.14	0.65	0.59	0.29
Lachnoanaerobaculum	0.92	1.34	0.66	1.45	-2.05	0.10	0.01	0.37	0.14	0.12	0.08	0.11
Haemophilus	2.10	1.53	3.66	-1.37	2.39	0.73	0.01	0.84	0.14	0.60	0.47	0.43
Pseudopropionibacterium	0.18	0.31	0.08	1.73	-3.80	0.74	0.02	0.84	0.16	0.99	0.60	0.30
Rothia	5.14	3.60	13.55	-1.43	3.76	0.83	0.02	0.91	0.16	0.36	0.52	0.32
Corynebacterium	6.73	8.62	5.63	1.28	-1.53	0.29	0.04	0.57	0.27	0.58	0.92	0.13
Actinomyces	15.50	11.24	7.91	-1.38	-1.42	0.10	0.07	0.37	0.39	0.05	0.06	0.14
Actinobaculum	1.67	3.83	1.82	2.30	-2.10	0.31	0.09	0.57	0.44	0.81	0.58	0.25
Neisseria	2.82	3.26	2.89	1.16	-1.13	0.19	0.64	0.52	0.89	0.20	0.11	0.01
Selenomonas	2.74	2.34	2.87	-1.17	1.23	0.32	0.77	0.57	0.91	0.25	0.40	0.20
Campylobacter	1.50	1.70	2.34	1.13	1.37	0.35	0.39	0.58	0.72	0.15	0.25	0.07
Saccharimonas	1.35	1.35	1.03	-1.00	-1.31	0.57	0.30	0.74	0.72	0.56	0.59	0.92
Gemella	0.83	1.76	1.40	2.12	-1.26	0.13	0.11	0.41	0.44	0.05	0.07	0.03
Tannerella	0.62	0.47	0.57	-1.33	1.21	0.19	0.70	0.52	0.91	0.18	0.15	0.08
Aggregatibacter	0.43	1.61	1.08	3.75	-1.49	0.11	0.21	0.40	0.67	0.10	0.05	0.02
Bergeyella	0.22	0.31	0.34	1.40	1.13	0.41	0.87	0.64	0.98	0.33	0.21	0.07
Eikenella	0.17	0.23	0.52	1.39	2.27	0.24	0.33	0.54	0.72	0.43	0.38	0.03
Peptostreptococcus	0.13	0.09	0.20	-1.36	2.08	0.27	1.00	0.57	1.00	0.07	0.12	0.42
Sphingomonas	0.13	0.00	0.00	-540.39	6.04	0.15	0.37	0.46	0.72	0.23	0.20	0.02
Acinetobacter	0.12	0.00	0.01	-501.19	49.73	0.33	0.18	0.57	0.63	0.84	0.76	0.09
Schwartzia	0.06	0.13	0.20	2.04	1.58	0.24	0.10	0.54	0.44	0.39	0.34	0.06
Phyllobacterium	0.03	0.00	0.00	-109.41	2.59	0.33	0.37	0.57	0.72	0.84	0.76	0.09
Halomonas	0.00	0.00	0.00	3.40	-3.40	0.18	0.37	0.51	0.72	0.04	0.05	0.07
Streptococcus	22.67	17.86	17.12	-1.27	-1.04	0.55	1.00	0.74	1.00	0.90	0.93	0.72

Veillonella	7.49	5.25	5.62	-1.43	1.07	0.22	0.47	0.54	0.76	0.25	0.26	0.39
Capnocytophaga	5.25	5.62	7.69	1.07	1.37	0.64	0.50	0.78	0.77	0.38	0.46	0.20
Prevotella	3.12	3.77	4.24	1.21	1.12	0.81	0.52	0.90	0.80	0.38	0.41	0.88

Table S3. Proteins Showed Significant Differences between High Bleeders vs. Low Bleeders at Baseline

ProteinID	Gene Names	Fold Change	*P* Value	Adjusted *p* Value	Disease Marker
Q9NRC6	SPTBN5	10.36	<0.0001	0.001	
P46940	IQGAP1	9.63	<0.0001	0.000	
P49411	TUFM	9.01	<0.0001	0.000	
Q05315	CLC	8.86	<0.0001	0.000	Yes
P01602	IGKV1-5	7.98	<0.0001	0.001	
P02751	FN1	6.96	0.008	0.035	Yes
O75367	MACROH2A1	6.39	<0.0001	0.000	
Q6P4A8	PLBD1	6.32	<0.0001	0.000	
A0A0C4DH35	IGHV3-35	5.65	<0.0001	0.001	
P68133	ACTA1	5.61	0.006	0.027	
P07305	H1-0	5.23	<0.0001	0.000	
Q15843	NEDD8	5.21	<0.0001	0.000	
Q9NP55	BPIFA1	4.73	0.000	0.005	
P55072	VCP	4.62	<0.0001	0.000	
P49643	PRIM2	4.58	0.000	0.003	
Q9NY33	DPP3	4.48	<0.0001	0.000	
P07900	HSP90AA1	4.39	<0.0001	0.000	
Q6XQN6	NAPRT	4.35	<0.0001	0.000	Yes
Q93077	H2AC6	4.31	0.001	0.007	
P11684	SCGB1A1	4.23	0.004	0.024	Yes
P05089	ARG1	4.21	<0.0001	0.000	Yes
Q14019	COTL1	4.11	0.009	0.037	
P01019	AGT	3.91	<0.0001	0.000	Yes
Q9H4A4	RNPEP	3.91	<0.0001	0.000	
Q9UJ70	NAGK	3.86	<0.0001	0.000	Yes
P26641	EEF1G	3.85	<0.0001	0.000	
P00352	ALDH1A1	3.79	<0.0001	0.001	Yes
Q13404	UBE2V1	3.74	<0.0001	0.000	
P19823	ITIH2	3.64	0.000	0.004	
O75368	SH3BGRL	3.59	<0.0001	0.002	
P05107	ITGB2	3.46	0.000	0.004	Yes
P14324	FDPS	3.44	<0.0001	0.000	
P58546	MTPN	3.44	<0.0001	0.001	Yes
P21333	FLNA	3.34	0.003	0.021	
P04179	SOD2	3.33	0.000	0.002	Yes
P62993	GRB2	3.28	<0.0001	0.002	
P04632	CAPNS1	3.26	<0.0001	0.000	
Q6ZN66	GBP6	3.25	0.001	0.007	Yes
P08174	CD55	3.21	<0.0001	0.001	Yes

P12814	ACTN1	3.16	0.000	0.003	
P27348	YWHAQ	3.11	<0.0001	0.000	Yes
O15511	ARPC5	3.05	0.005	0.025	
O75594	PGLYRP1	3.02	0.002	0.011	Yes
P02760	AMBP	3.01	<0.0001	0.000	Yes
P62879	GNB2	2.93	0.001	0.011	
P20700	LMNB1	2.90	0.000	0.003	Yes
P68104	EEF1A1	2.89	0.006	0.027	
P25774	CTSS	2.88	0.002	0.012	Yes
P06753	TPM3	2.86	0.002	0.014	Yes
P08670	VIM	2.86	0.005	0.025	Yes
P17213	BPI	2.79	0.027	0.081	
P80723	BASP1	2.79	0.002	0.012	
Q15365	PCBP1	2.77	<0.0001	0.000	
A0A0C4DH36	IGHV3-38	2.76	0.001	0.009	
P00491	PNP	2.76	0.024	0.075	
P25815	S100P	2.76	0.005	0.025	Yes
Q04695	KRT17	2.75	0.001	0.009	Yes
P48595	SERPINB10	2.73	<0.0001	0.001	
Q969H8	MYDGF	2.73	<0.0001	0.001	Yes
O15144	ARPC2	2.72	0.001	0.011	
Q04917	YWHAH	2.71	0.001	0.007	
A0A075B6H9	IGLV4-69	2.70	0.001	0.008	
P04040	CAT	2.68	<0.0001	0.002	Yes
Q9NZH8	IL36G	2.68	0.001	0.008	Yes
P08637	FCGR3A	2.66	0.003	0.016	
P12429	ANXA3	2.64	0.019	0.062	Yes
P01023	A2M	2.61	0.001	0.011	
P06737	PYGL	2.60	0.004	0.024	
P16070	CD44	2.60	0.002	0.013	
P39687	ANP32A	2.58	0.001	0.006	
P04259	KRT6B	2.56	0.020	0.064	
P31146	CORO1A	2.56	0.004	0.022	Yes
P02679	FGG	2.55	0.001	0.011	
P02753	RBP4	2.55	0.002	0.012	
P61158	ACTR3	2.54	0.001	0.007	
P01861	IGHG4	2.53	0.015	0.053	
P32320	CDA	2.52	0.003	0.016	Yes
O15143	ARPC1B	2.51	0.007	0.030	
P53004	BLVRA	2.51	0.003	0.018	
Q9UM07	PADI4	2.51	0.003	0.020	Yes
O75083	WDR1	2.50	0.002	0.012	
P00390	GSR	2.50	0.001	0.009	Yes
P01008	SERPINC1	2.50	<0.0001	0.001	Yes
P41218	MNDA	2.50	0.016	0.054	Yes
P61088	UBE2N	2.49	0.001	0.006	
P14780	MMP9	2.46	0.007	0.030	Yes

P27797	CALR	2.46	0.007	0.033	
Q86T26	TMPRSS11B	2.46	0.001	0.009	Yes
Q14624	ITIH4	2.45	<0.0001	0.001	Yes
P05164	MPO	2.42	0.020	0.064	Yes
P02671	FGA	2.40	0.001	0.009	Yes
Q92820	GGH	2.39	0.002	0.014	Yes
P35754	GLRX	2.38	0.008	0.033	Yes
P43652	AFM	2.38	0.002	0.012	Yes
A0A0A0MT36	IGKV6D-21	2.37	0.009	0.036	
P61956	SUMO2	2.37	0.014	0.049	
Q6UX06	OLFM4	2.36	0.045	0.116	Yes
P50552	VASP	2.35	0.001	0.011	Yes
P16403	H1-2	2.34	0.005	0.026	
P18206	VCL	2.32	0.003	0.018	
Q99880	H2BC13	2.32	0.043	0.114	
P17900	GM2A	2.31	<0.0001	0.002	Yes
Q13231	CHIT1	2.30	0.009	0.036	Yes
P22314	UBA1	2.28	0.009	0.037	
P52566	ARHGDIB	2.28	0.029	0.086	
P62805	H4C1; H4C2; H4C3; H4C4; H4C5; H4C6; H4C8; H4C9; H4C11; H4C12; H4C13; H4C14; H4C15; H4C16	2.26	0.030	0.088	
P09960	LTA4H	2.25	0.005	0.025	Yes
P49720	PSMB3	2.25	0.004	0.024	
Q9BPY8	HOPX	2.25	0.008	0.034	
P60660	MYL6	2.22	0.007	0.030	
P26583	HMGB2	2.21	0.011	0.042	
Q9BRF8	CPPED1	2.21	0.014	0.050	Yes
P02766	TTR	2.20	0.006	0.030	Yes
P22894	MMP8	2.18	0.031	0.088	Yes
P60953	CDC42	2.18	0.019	0.062	
Q9UL46	PSME2	2.16	0.001	0.008	Yes
P00738	HP	2.14	0.005	0.025	
Q86UX7	FERMT3	2.13	0.007	0.032	
P20618	PSMB1	2.11	0.014	0.050	
P60900	PSMA6	2.11	0.035	0.096	
P61981	YWHAG	2.11	0.035	0.096	
P00734	F2	2.10	0.005	0.026	Yes
P02675	FGB	2.10	0.002	0.015	
P08603	CFH	2.08	0.007	0.033	Yes
P31151	S100A7	2.06	0.010	0.040	
P80748	IGLV3-21	2.06	0.007	0.030	
P28676	GCA	2.05	0.010	0.038	
P02647	APOA1	2.04	0.019	0.063	Yes
P13639	EEF2	2.03	0.035	0.096	

P30101	PDIA3	2.03	0.009	0.035	Yes
O00764	PDXK	2.02	0.007	0.033	
O75874	IDH1	2.01	0.011	0.040	
P11413	G6PD	2.01	0.023	0.072	
P43490	NAMPT	2.00	0.028	0.084	Yes
Q01518	CAP1	2.00	0.002	0.015	
O75223	GGCT	1.99	0.005	0.025	Yes
P01042	KNG1	1.99	0.008	0.035	
P04217	A1BG	1.97	0.023	0.073	Yes
P15153	RAC2	1.97	0.044	0.115	
P36871	PGM1	1.97	0.040	0.107	
P04004	VTN	1.95	0.037	0.100	
P00747	PLG	1.94	0.003	0.019	Yes
P30520	ADSS2	1.92	0.006	0.027	
Q9Y490	TLN1	1.91	0.034	0.096	
P07737	PFN1	1.87	0.001	0.006	
P07741	APRT	1.87	0.031	0.089	Yes
P61160	ACTR2	1.87	0.040	0.107	
P14550	AKR1A1	1.86	0.015	0.053	
P25786	PSMA1	1.85	0.016	0.054	Yes
P27695	APEX1	1.85	0.020	0.064	Yes
P49913	CAMP	1.84	0.013	0.046	
P13796	LCP1	1.83	0.004	0.022	Yes
P31946	YWHAB	1.82	0.019	0.063	
P19957	PI3	1.80	0.027	0.080	Yes
P22626	HNRNPA2B1	1.80	0.012	0.043	
P52790	HK3	1.80	0.015	0.052	
Q14974	KPNB1	1.79	0.037	0.101	
P59998	ARPC4	1.74	0.001	0.007	
P13693	TPT1	1.73	0.028	0.084	Yes
P26447	S100A4	1.73	0.032	0.091	Yes
P01859	IGHG2	1.71	0.025	0.077	
P42785	PRCP	1.71	0.010	0.039	Yes
P52907	CAPZA1	1.68	0.035	0.096	
Q6ZMR5	TMPRSS11A	1.68	0.050	0.127	
P29401	TKT	1.66	0.003	0.019	
P61019	RAB2A	1.64	0.026	0.078	
P61026	RAB10	1.63	0.031	0.088	Yes
P14618	PKM	1.57	0.004	0.024	
P01857	IGHG1	1.56	0.005	0.026	
P06744	GPI	1.54	0.002	0.012	Yes
P02790	HPX	1.53	0.024	0.075	
P26038	MSN	1.53	0.011	0.040	
P01860	IGHG3	1.52	0.034	0.096	
P35321	SPRR1A	-1.56	0.045	0.116	
P03973	SLPI	-1.59	0.018	0.061	
P12273	PIP	-1.59	0.024	0.075	

Q9UGM3	DMBT1	-1.59	0.025	0.078	
Q9UBC9	SPRR3	-1.64	0.009	0.037	Yes
Q9UBX7	KLK11	-1.67	0.033	0.092	Yes
P61626	LYZ	-1.96	0.010	0.039	
Q8TAX7	MUC7	-2.08	0.005	0.026	
P28072	PSMB6	-2.17	0.010	0.040	
Q8NBJ4	GOLM1	-2.22	0.046	0.117	
Q96DA0	ZG16B	-2.27	0.002	0.011	
O43490	PROM1	-2.44	0.005	0.026	
Q9UKR0	KLK12	-2.86	0.005	0.026	Yes
P15516	HTN3	-3.13	0.009	0.037	
P01011	SERPINA3	-3.70	0.005	0.026	Yes
P10163	PRB4	-3.70	0.003	0.018	
A0A0G2JRQ6		-4.76	0.001	0.008	
P0DP25	CALM3	-5.26	<0.0001	0.000	
A0A075B6S2	IGKV2D-29	-7.69	0.001	0.008	

Table S4. GO Biological Process Showed Significant Differences between High Bleeders vs. Low Bleeders at Baseline

Downregulated in High Bleeders		
**GO Biological Process**	FDR *P* value	Genes
**Tissue homeostasis**	2.98E-04	LYZ PIP ZG16B PROM1 SERPINA3
**Antimicrobial humoral response**	3.90E-04	SLPI MUC7 LYZ DMBT1
**Multicellular organismal homeostasis**	2.88E-03	LYZ PIP ZG16B PROM1 SERPINA3
**Humoral immune response**	3.54E-03	SLPI MUC7 LYZ DMBT1
**Defense response to bacterium**	5.86E-03	LYZ SLPI HTN3 DMBT1
Upregulated in High Bleeders		
**GO Biological Process**	FDR P value	Genes
**Immune system process**	7.93E-17	BPI PI3 RAC2 IL36G CTSS MNDA CAMP GBP6 CD55 BPIFA1 FCGR3A FLNA CFH PGLYRP1 CD44 HSP90AA1 GPI FN1 APOA1 ARG1 LCP1 ANXA3 S100A7 MSN H4C8 ITGB2 PDIA3 FGA FGB A2M F2 H4C3 H4C11 PNP H4C15 H4C14 H4C12 H4C6 H4C13 H4C9 H4C5 H4C4 H4C1 H4C2 MPO VIM CDC42 WDR1 MMP9 CORO1A VTN HPX SOD2 ACTR3 MMP8 PLG PSMA1 ACTR2 SCGB1A1 PADI4 HMGB2 EEF2 CALR HP ACTN1 RAB10 CLC G6PD UBE2N RBP4 ADSS2 AMBP CHIT1
**Defense response**	4.70E-08	MPO PGLYRP1 CD44 BPI PI3 IL36G MNDA CAMP GBP6 BPIFA1 HP PSMA6 CFH HSP90AA1 APOA1 ANXA3 HMGB2 FGA FGB A2M F2 FCGR3A VIM ITIH4 CDC42 COTL1 HPX KNG1 ACTR3 FN1 MMP8 ARG1 PSMA1 PRCP ACTR2 SCGB1A1 PADI4 ITGB2 CD55 CORO1A S100A7 MMP9 AGT
**Response to stress**	4.75E-08	VCL TLN1 FERMT3 FGA FLNA MPO PGLYRP1 CD44 HSP90AA1 APEX1 BPI GSR KNG1 FN1 CAT PI3 UBA1 IL36G PRCP MNDA CAMP VCP PDIA3 FGG FGB UBE2N CALR F2 GBP6 BPIFA1 UBE2V1 HP PSMA6 VTN CFH MMP9 APOA1 PLG ACTR2 ANXA3 S100A7 HMGB2 A2M FCGR3A VIM ITIH4 CDC42 COTL1 GPI HPX SOD2 MACROH2A1 ACTR3 SERPINC1 MMP8 ARG1 PSMA1 AGT IDH1 SCGB1A1 PADI4 ITGB2 CD55 CORO1A TPT1 NAPRT G6PD GRB2 AMBP

**Response to other organism**	4.75E-07	MPO PGLYRP1 BPI PI3 IL36G MNDA CAMP GBP6 BPIFA1 CFH HSP90AA1 ANXA3 HMGB2 FGA FGB A2M F2 CD55 FCGR3A VIM CDC42 PYGL COTL1 HPX SOD2 ACTR3 ARG1 ACTR2 SCGB1A1 PADI4 CALR HP TPT1 S100A7 EEF1G CORO1A CHIT1
**Inflammatory response**	3.36E-04	CD44 IL36G HP PSMA6 APOA1 F2 FCGR3A PGLYRP1 ITIH4 KNG1 FN1 MMP8 PSMA1 PRCP SCGB1A1 ITGB2 HMGB2 A2M MMP9 AGT
**Response to oxidative stress**	3.63E-04	GSR CAT MMP9 S100A7 HP MPO APEX1 SOD2 MACROH2A1 ARG1 IDH1 SCGB1A1 NAPRT G6PD
**Wound healing**	9.30E-05	VCL TLN1 FERMT3 FGA FLNA KNG1 FN1 PRCP FGG FGB F2 VTN PLG SERPINC1 CD44

Table S5. Example Proteins with Significant Differences in this Study and Their Disease Association from "Atlas of the plasma proteome" [27]

Protein	Disease	P value	OR (odds ratio >1, Positive Association)	NB individual	NB case
**APEX1**	Soft tissue disorders related to use, overuse and pressure	0.00	1.71	24962	58
**APEX1**	Other respiratory disorders and diseases	0.00	1.66	27646	86
**APEX1**	Smoking dependency	0.00	1.63	26468	69
**APEX1**	Palmar fascial fibromatosis [Dupuytren]	0.03	1.52	24952	48
**APEX1**	Alcohol use disorder, ICD-based	0.00	1.51	28135	165
**APEX1**	ILD, hospital admissions	0.04	1.51	28376	43
**APEX1**	Interstitial lung disease	0.05	1.49	27155	43
**APEX1**	Glaucoma	0.05	1.46	28121	52
**APEX1**	Hyperlipidaemia, other/unspecified	0.02	1.45	24361	76
**APEX1**	Other diseases of oesophagus	0.03	1.44	22774	66
**APEX1**	Other respiratory diseases principally affecting the interstitium	0.05	1.43	28313	57
**APEX1**	Alcohol dependence	0.02	1.40	26503	100
**APEX1**	GI-bleeding	0.00	1.40	27105	177
**APEX1**	Other diseases of the digestive system	0.00	1.39	27110	182
**APEX1**	Other diseases of pleura	0.00	1.38	27234	183
**APEX1**	Other disorders of fluid, electrolyte and acid-base balance	0.04	1.37	24365	80
**APEX1**	Influenza and pneumonia	0.00	1.36	27279	191
**APEX1**	Substance abuse, excluding more controls	0.00	1.36	24270	301
**APEX1**	Implicit bacterial Sepsis	0.01	1.35	25741	146
**APEX1**	Heart failure, strict	0.02	1.34	27988	122
**APEX1**	HSIL lesion of the cervix uteri	0.02	1.34	15754	113

**APEX1**	Mental and behavioural disorders due to alcohol, excluding acute intoxication	0.00	1.34	28063	231
**APEX1**	Pneumonia, organism unspecified	0.01	1.34	27250	162
**APEX1**	HSIL lesion of the cervix uteri, vagina or vulva	0.02	1.33	15745	122
**APEX1**	Diseases of liver	0.03	1.32	27750	125
**APEX1**	Ulcer of oesophagus	0.02	1.32	22841	133
**APEX1**	Other sleep disorders	0.02	1.30	26814	174
**APEX1**	Urolithiasis	0.00	1.29	28121	246
**APEX1**	Condition for Implicit Sepsis (Organ dysfunction codes)	0.00	1.28	26118	366
**APEX1**	Metabolic disorders	0.00	1.28	24997	712
**APEX1**	Other disorders of bladder	0.03	1.27	26246	185
**APEX1**	Calculus of kidney and ureter	0.04	1.26	28047	172
**APEX1**	Disorders of lipoprotein metabolism and other lipidaemias	0.00	1.26	24831	546
**APEX1**	All dysplastic lesions of the cervix uteri, vagina or vulva	0.04	1.23	15724	205
**APEX1**	Implicit Sepsis	0.00	1.23	20449	504
**APEX1**	Other intervertebral disc disorders	0.02	1.23	25693	253
**APEX1**	Type 2 diabetes	0.01	1.23	26875	334
**APEX1**	Type 2 diabetes without complications	0.02	1.23	26865	324
**APEX1**	All dysplastic lesions of the cervix uteri	0.05	1.22	15734	197
**APEX1**	Pure hypercholesterolaemia	0.00	1.22	24785	500
**APEX1**	Hypertension, essential	0.00	1.20	23385	1325
**APEX1**	Hypertension	0.00	1.19	23410	1350
**APEX1**	Death due to cardiac causes	0.00	1.16	22309	1996
**APEX1**	Diseases of the respiratory system	0.00	1.13	24126	1978
**APEX1**	Chronic lower respiratory diseases	0.03	1.12	26096	891
**APEX1**	Noninfective enteritis and colitis	0.04	1.12	27602	761
**CD55**	Disorders of parathyroid gland	0.00	8.13	49203	263
**CD55**	Type 2 diabetes with renal complications	0.00	6.74	4023	106
**CD55**	Diabetic nephropathy, excluding more controls	0.00	6.58	4033	114
**CD55**	Hypertensive Renal Disease	0.00	3.71	32899	272
**CD55**	Type 2 diabetes with peripheral circulatory complications	0.00	3.71	45075	119
**CD55**	Dialysis	0.00	3.59	44803	160
**CD55**	Chronic kidney disease	0.00	3.39	47621	2978
**CD55**	Unspecified kidney failure	0.00	3.39	44899	256
**CD55**	Type 2 diabetes with neurological complications	0.00	3.18	4083	164
**CD55**	Hyperkalaemia	0.00	3.17	38434	572
**CD55**	Diabetic neuropathy	0.00	3.16	4884	193
**CD55**	Hyperparathyroidism	0.00	3.04	49188	248
**CD55**	Recurrent and persistent haematuria	0.00	3.01	49240	94
**CD55**	Other disorders of glucose regulation and pancreatic internal secretion	0.00	2.99	49700	446
**CD55**	Hypertension, portal	0.00	2.90	49389	150
**CD55**	Oesophageal varices	0.00	2.89	46642	106
**CD55**	Other other unspecified disorders of the circulatory system	0.00	2.89	47275	110
**CD55**	Chronic nephritic syndrome	0.00	2.85	49308	162
**CD55**	Pulmonary oedema	0.00	2.74	48908	129

**CD55**	Acute peritonitis	0.00	2.68	48812	181
**CD55**	Arterial embolism and thrombosis of lower extremity artery	0.00	2.67	47363	112
**CD55**	Fluid overload	0.00	2.65	38363	501
**CD55**	Other specified disorders of kidney and ureter	0.00	2.65	48960	310
**CD55**	Cirrhosis of liver, NAS	0.00	2.64	49446	207
**CD55**	Diseases of spleen	0.00	2.62	48639	137
**CD55**	Fibrosis and cirrhosis of liver	0.00	2.56	48093	249
**CD55**	Renal tubulo-interstitial diseases	0.00	2.54	49234	5170
**CD55**	Acute renal failure	0.00	2.53	47669	3026
**CD55**	Disorders of plasma-protein metabolism, not elsewhere classified	0.00	2.51	37976	114
**CD55**	All influenza	0.00	2.48	45828	228
**CD55**	Other diseases of blood and blood-forming organs	0.00	2.48	48628	126
**CD55**	Other and unspecified aplastic anaemias	0.00	2.47	46032	165
**CD55**	All influenza (not pneumonia)	0.00	2.45	45823	223
**CD55**	Other arterial embolism and thrombosis	0.00	2.44	47446	195
**CD55**	Alcoholic liver disease	0.00	2.37	47990	146
**CD55**	Implicit fungal Sepsis	0.00	2.34	43747	500
**CD55**	Bacterial pneumonia (organism specified)	0.00	2.33	48936	270
**CD55**	Folate deficiency anaemia	0.00	2.33	46878	97
**CD55**	Iron deficiency	0.00	2.32	44763	135
**CD55**	Acidosis	0.00	2.31	38475	613
**CD55**	Other infectious diseases	0.00	2.31	49724	98
**CD55**	Type 1 diabetes without complications	0.00	2.31	45251	295
**CD55**	Hepatic failure, not elsewhere classified	0.00	2.29	47969	125
**CD55**	Implicit viral Sepsis	0.00	2.27	43445	749
**CD55**	Atherosclerosis, excluding cerebral and coronary sclerosis	0.00	2.25	47629	378
**CD55**	Glomerular disorders in diseases classified elsewhere	0.00	2.25	49306	160
**CD55**	Bipolar affective disorders	0.00	2.24	46166	135
**CD55**	Viral hepatitis	0.00	2.23	49681	105
**CD55**	Gout	0.00	2.22	35524	1154
**CD55**	Other disorders of kidney and ureter	0.00	2.22	49587	937
**CD55**	!Phlebitis/thrombophlebitis venae iliacae [externae/internae/communis], drug-induced	0.00	2.21	49509	475
**CD55**	Disorders of iron metabolism	0.00	2.19	37983	121
**CD55**	Tubulo-interstitial nephritis, not specified as acute or chronic	0.00	2.17	44296	232
**CD55**	Other diseases of pancreas	0.00	2.16	46090	292
**CD55**	Bacterial pneumonia, not elsewhere classified	0.00	2.15	45757	157
**CD55**	Restless leg syndrome	0.00	2.14	49722	150
**CD55**	Other and unspecified polyneuropathies, also in other diseases	0.00	2.13	49575	570
**CD55**	Acute upper respiratory infections of multiple and unspecified sites	0.00	2.12	49234	124
**CD55**	DVT of lower extremities	0.00	2.12	47049	513
**CD55**	Osteomyelitis	0.00	2.11	45831	139
**CD55**	Crohn's disease NAS	0.00	2.10	46501	172

**CD55**	Enterocolitis due to Clostridium difficile	0.00	2.10	49298	169
**CD55**	Gout, unspecified	0.00	2.09	49573	1138
**CD55**	Peripheral artery disease	0.00	2.09	47966	773
**CD55**	Viral warts	0.00	2.08	49398	100
**CD55**	Nontoxic multinodular goitre	0.00	2.06	45777	91
**CD55**	Ulcer of lower limb, not elsewhere classified	0.00	2.03	45932	450
**CD55**	Other and unspecified epidermal thickening	0.01	2.02	45605	123
**CD55**	Other diseases of arteries and capillaries	0.00	2.02	47713	462
**CD55**	Disorders of other endocrine glands	0.00	1.99	49570	630
**CD55**	Thrombocytopenia, unspecified	0.00	1.96	49262	448
**CD55**	Implicit bacterial Sepsis	0.00	1.95	43980	2134
**CD55**	Inflammatory diseases of the central nervous system	0.00	1.93	49689	134
**CD55**	Type 2 diabetes with ophthalmic complications	0.00	1.93	4350	485
**CD55**	Sequelae of cerebrovascular disease	0.00	1.90	47203	373
**CD55**	Crohn disease	0.00	1.88	46510	181
**CD55**	Other obstructive and reflux uropathy	0.00	1.87	44721	657
**CD55**	Cerebral atherosclerosis	0.04	1.86	49720	97
**CD55**	Chronic pancreatitis	0.03	1.86	45896	98
**CD55**	Hyperosmolality and hypernatraemia	0.00	1.86	38090	228
**CD55**	Other specified/unspecified disorders of synovium and tendon +Other specified/unspecified bursopathies	0.02	1.86	42578	104
**CD55**	Other epidermal thickening	0.02	1.85	45613	131
**CD55**	Other viral diseases	0.00	1.85	49572	303
**CD55**	Pruritus	0.01	1.85	48737	135
**CD55**	Purpura and other haemorrhagic conditions	0.00	1.85	49357	543
**CD55**	Acute gastritis	0.00	1.84	37419	175
**CD55**	Cervicalgia	0.00	1.84	43136	349
**CD55**	Hypertension, Pulmonary Arterial	0.00	1.83	32969	342
**CD55**	Malignant neoplasm of intrahepatic ducts, biliary tract and gallbladder, excluding all cancers (controls excluding all cancers)	0.01	1.83	39647	157
**CD55**	Other disorders of eye and adnexa	0.00	1.83	49681	296
**CD55**	Other disorders of the genitourinary system	0.01	1.83	49649	132
**CD55**	Small fibre neuropathy	0.04	1.82	49101	96
**CD55**	Background diabetic retinopathy	0.00	1.81	49385	446
**CD55**	Chondropathies	0.00	1.80	49241	177
**CD55**	Other coagulation defects	0.00	1.80	49035	221
**CD55**	Phlebitis and thrombophlebitis (not including DVT)	0.00	1.80	47209	673
**CD55**	Cardiac arrest	0.00	1.79	27513	366
**CD55**	Idiopathic thrombocytopenic purpura	0.05	1.78	48911	97
**CD55**	Labyrinthitis	0.03	1.78	49256	110
**CD55**	Disorder of thyroid, unspecified	0.01	1.77	45837	151
**CD55**	Disorders of calcium metabolism	0.00	1.76	38409	547
**CD55**	Other disorders of veins	0.01	1.76	46719	183
**CD55**	Other extrapyramidal and movement disorders in other diseases	0.00	1.76	48793	323
**CD55**	Other aneurysm	0.02	1.75	47401	150
**CD55**	Other bursopathies	0.01	1.75	42658	184

**CD55**	Other viral diseases, not elsewhere classified	0.00	1.75	49534	265
**CD55**	Malignant neoplasm of kidney, except renal pelvis, excluding all cancers (controls excluding all cancers)	0.01	1.74	39699	209
**CD55**	Venous thromboembolism	0.00	1.74	49293	1455
**CD55**	Volume depletion	0.00	1.74	39336	1474
**CD55**	Bacterial infection, other or unspecified	0.00	1.73	47724	293
**CD55**	Other contact dermatitis	0.03	1.73	48730	128
**CD55**	Pulmonary embolism	0.00	1.73	48799	915
**CD55**	Explicit Sepsis	0.00	1.72	45526	1866
**CD55**	Streptococcus and staphylococcus as the cause of diseases classified to other chapters	0.00	1.72	47071	812
**CD55**	Neuralgia and neuritis, unspecified	0.05	1.71	42591	117
**CD55**	Other bacterial agents as the cause of diseases classified to other chapters	0.00	1.71	48053	1794
**CD55**	Postprocedural musculoskeletal disorders, not elsewhere classified	0.05	1.69	49596	126
**CD55**	Crohn disease (strict definition, all UC cases excluded)	0.04	1.68	49041	133
**CD55**	Hydronephrosis	0.00	1.67	44627	563
**CD55**	Other necrotizing vasculopathies	0.03	1.67	48548	177
**CD55**	Other peripheral vascular diseases	0.00	1.67	48287	1036
**CD55**	Other bacterial diseases	0.00	1.66	49494	2063
**CD55**	Other septicaemia	0.00	1.66	49232	1801
**CD55**	Pneumonitis due to solids and liquids	0.00	1.66	49581	511
**CD55**	Viral and other specified intestinal infections	0.02	1.65	46831	212
**CD55**	Other disorders of fluid, electrolyte and acid-base balance	0.00	1.64	40911	3049
**CD55**	Other respiratory diseases principally affecting the interstitium	0.00	1.63	49551	772
**CD55**	Cellulitis	0.00	1.62	48477	1355
**CD55**	Myocardial infarction, with ST-elevation	0.01	1.62	49550	299
**CD55**	Pain in joint	0.00	1.62	39327	1444
**CD55**	Candidiasis	0.00	1.61	49477	827
**CD55**	Hemiplegia	0.00	1.61	49440	490
**CD55**	Hypotension	0.00	1.61	49442	2277
**CD55**	Other inflammatory liver diseases	0.03	1.61	48036	192
**CD55**	Panic disorder	0.02	1.61	46922	199
**CD55**	Myocardial infarction, strict	0.00	1.58	44482	1359
**CD55**	Other disorders of male genital organs	0.01	1.58	18807	292
**CD55**	Unspecified acute lower respiratory infection	0.00	1.58	49205	1824
**CD55**	Decubitus ulcer and pressure area	0.00	1.57	46269	787
**CD55**	Other acute lower respiratory infections	0.00	1.57	49248	1867
**CD55**	Hallux valgus (acquired)	0.00	1.56	34997	627
**CD55**	Ischaemic Stroke, excluding all haemorrhages	0.00	1.56	48987	1074
**CD55**	Corneal degeneration	0.02	1.55	49220	283
**CD55**	Implicit Sepsis	0.00	1.55	34733	3823
**CD55**	Other anaemias	0.00	1.55	48798	2931
**CD55**	Other and unspecified anaemias	0.00	1.55	48797	2930
**CD55**	Condition for Implicit Sepsis (Organ dysfunction codes)	0.00	1.54	48176	6171
**CD55**	Disorders of mineral metabolism	0.00	1.54	38943	1081

**CD55**	Other and unspecified local infections of skin and subcutaneous tissue	0.01	1.54	47495	373
**CD55**	Viral pneumonia	0.00	1.54	46076	476
**CD55**	Abdominal aortic aneurysm (AAA)	0.02	1.53	47566	315
**CD55**	Other specified/unspecified soft tissue disorders	0.00	1.53	43531	1057
**CD55**	Viral infections characterized by skin and mucous membrane lesions	0.02	1.53	49593	295
**CD55**	Mycoses	0.00	1.52	49629	979
**CD55**	Other and unspecified degenerative diseases of nervous system	0.01	1.51	49050	393
**CD55**	Bacterial, viral and other infectious agents	0.00	1.50	48931	2672
**CD55**	Coxarthrosis, primary	0.01	1.50	49463	359
**CD55**	Hypo-osmolality and hyponatraemia	0.00	1.50	39015	1153
**CD55**	Influenza and pneumonia	0.00	1.50	49290	3690
**CD55**	Myocardial infarction, without ST-elevation	0.00	1.50	49666	591
**CD55**	Infections of the skin and subcutaneous tissue	0.00	1.49	48961	1839
**CD55**	Noninfectious colitis NAS	0.00	1.49	47779	1450
**CD55**	Pleural effusion	0.00	1.49	47340	1918
**CD55**	Lung diseases due to external agents	0.00	1.48	49703	633
**CD55**	Other noninfective disorders of lymphatic vessels and lymph nodes	0.04	1.48	46797	261
**CD55**	Pneumonia, organism unspecified	0.00	1.47	48804	3204
**CD55**	Radiation-related disorders of the skin and subcutaneous tissue	0.00	1.47	49622	633
**CD55**	Actinic keratosis	0.01	1.46	49566	577
**CD55**	Bacterial intestinal infections, IBD co-morbidities	0.02	1.46	49438	384
**CD55**	Other noninfective gastroenteritis and colitis	0.00	1.46	48020	1691
**CD55**	Secondary right heart disease	0.00	1.46	48540	656
**CD55**	Duodenal ulcer	0.02	1.45	37633	389
**CD55**	Epilepsy	0.01	1.45	46718	557
**CD55**	Left bundle-branch block	0.00	1.45	41761	690
**CD55**	Other bacterial intestinal infections	0.02	1.45	47005	386
**CD55**	Other pulmonary heart/vessel disease	0.00	1.45	48550	666
**CD55**	Other specified cerebrovascular diseases, other cerebrovascular disorders in diseases classified elsewhere	0.00	1.45	48012	1182
**CD55**	Cerebral palsy and other paralytic syndromes	0.00	1.44	49570	620
**CD55**	Fibromyalgia	0.01	1.44	42899	425
**CD55**	Interstitial lung disease	0.00	1.44	47325	619
**CD55**	Gastric ulcer	0.00	1.43	37943	699
**CD55**	Other arrhythmias	0.00	1.43	41888	817
**CD55**	Rheumatoid arthritis	0.00	1.43	35252	882
**CD55**	Skin changes due to chronic exposure to nonionizing radiation	0.01	1.43	49601	612
**CD55**	Vascular disorders of the intestines	0.05	1.43	35353	333
**CD55**	Visual impairment including blindness (binocular or monocular)	0.04	1.43	48751	332
**CD55**	Heart failure, strict	0.00	1.42	49319	2114
**CD55**	Idiopathic pulmonary fibrosis	0.01	1.42	47257	551

**CD55**	Diarrhoea and gastroenteritis of presumed infectious origin	0.00	1.41	48986	2367
**CD55**	Disorders of vitreous body	0.05	1.41	49546	338
**CD55**	Stroke, including SAH	0.00	1.41	48926	1308
**CD55**	Delirium, not induced by alcohol and other psychoactive substances	0.00	1.40	48825	1098
**CD55**	Other/unspecified dorsalgia	0.00	1.40	43890	1103
**CD55**	Stroke, excluding SAH	0.00	1.40	48965	1232
**CD55**	Viral pneumonia (unknown virus, not influenza)	0.04	1.40	48966	392
**CD55**	Cholecystitis	0.05	1.39	46193	395
**CD55**	Major coronary heart disease event	0.00	1.39	48799	1606
**CD55**	Ulcer of oesophagus	0.02	1.39	37756	512
**CD55**	Bradycardia, unspecified, drug-induced	0.00	1.38	49608	822
**CD55**	Cyst of kidney	0.02	1.38	49236	586
**CD55**	Emphysema	0.02	1.38	43019	602
**CD55**	Intestinal infectious diseases	0.00	1.38	49409	2790
**CD55**	Noninfective enteritis and colitis	0.00	1.38	48259	1930
**CD55**	Gastroduodenal ulcer	0.00	1.37	38287	1043
**CD55**	Other diseases of pleura	0.00	1.37	49265	3843
**CD55**	Other joint disorders	0.00	1.37	38497	4127
**CD55**	Urethral stricture	0.05	1.37	43790	421
**CD55**	Diseases of the blood and blood-forming organs and certain disorders involving the immune mechanism	0.00	1.36	48202	5603
**CD55**	Low back pain	0.00	1.36	44007	1220
**CD55**	Other diseases of liver	0.00	1.36	49188	1344
**CD55**	Other disorders of bone density and structure	0.01	1.36	46425	733
**CD55**	Other disorders of urethra and urinary system	0.00	1.36	46712	3343
**CD55**	Other respiratory disorders and diseases	0.00	1.36	49516	2501
**CD55**	Mood disorders, excluding more controls	0.00	1.35	43810	3061
**CD55**	Other/unspecified rheumatoid arthritis	0.01	1.35	49478	845
**CD55**	Polyarthropathies	0.00	1.35	39149	4779
**CD55**	Sciatica+with lumbago	0.04	1.35	43285	498
**CD55**	Agranulocytosis	0.02	1.34	49155	653
**CD55**	Certain infectious and parasitic diseases	0.00	1.34	47980	6791
**CD55**	Diseases of liver	0.00	1.34	49488	1644
**CD55**	Other (seronegative) rheumatoid arthritis, wide	0.01	1.34	49455	870
**CD55**	Depression	0.00	1.33	49037	3006
**CD55**	Disorders of gallbladder, biliary tract and pancreas	0.00	1.33	48516	2718
**CD55**	Genitourinary diseases	0.00	1.33	41513	10363
**CD55**	Other soft tissue disorders, not elsewhere classified	0.00	1.33	44859	2385
**CD55**	Vitamin D deficiency	0.02	1.33	45331	703
**CD55**	Disorders of choroid and retina	0.00	1.32	49299	1996
**CD55**	Other specified/unspecified hearing loss	0.00	1.32	49052	1238
**CD55**	Disorders of the thyroid gland	0.00	1.31	48687	3001
**CD55**	Varicose veins	0.02	1.31	47355	819
**CD55**	Depression or dysthymia	0.00	1.30	43321	2911
**CD55**	Rheumatic valve diseases	0.00	1.30	49590	1265
**CD55**	Smoking dependency	0.00	1.30	48702	2631

**CD55**	Oesophagitis	0.01	1.28	38412	1168
**CD55**	Other disorders of skin and subcutaneous tissue	0.00	1.28	48619	3137
**CD55**	Other specified/unspecified arthritis	0.00	1.28	37483	3113
**CD55**	Barrett oesophagus	0.04	1.27	38079	835
**CD55**	Dermatitis and eczema	0.03	1.26	49526	924
**CD55**	Other disorders of ear	0.00	1.26	49532	1718
**CD55**	Diseases of peritoneum	0.04	1.25	49556	925
**CD55**	Other diseases of the digestive system	0.00	1.25	48656	2268
**CD55**	Falls/tendency to fall	0.00	1.24	48583	4934
**CD55**	GI-bleeding	0.00	1.24	48626	2238
**CD55**	Osteopathies and chondropathies	0.00	1.24	48967	3275
**CD55**	Other or ill-defined heart diseases	0.00	1.24	43055	1984
**CD55**	Substance abuse, excluding more controls	0.00	1.24	43887	3138
**CD55**	Arthropathies	0.00	1.22	45264	10894
**CD55**	Other diseases of stomach and duodenum	0.01	1.22	39087	1843
**CD55**	Other diseases of urinary system	0.00	1.22	47702	4333
**CD55**	Spondylosis	0.01	1.22	44766	1979
**CD55**	Cholelithiasis	0.02	1.21	47718	1920
**CD55**	Gonarthrosis	0.00	1.21	37634	3264
**CD55**	Other arthrosis	0.00	1.21	39181	4811
**CD55**	Other malignant neoplasms of skin (=non-melanoma skin cancer), excluding all cancers (controls excluding all cancers)	0.01	1.21	41553	2063
**CD55**	Diseases of the skin and subcutaneous tissue	0.00	1.20	46725	6180
**CD55**	Other dorsopathies, not elsewhere classified	0.00	1.20	46286	3499
**CD55**	Squamous cell neoplasms and carcinomas of the skin, excluding all cancers (controls excluding all cancers)	0.01	1.20	41660	2170
**CD55**	Coxarthrosis	0.03	1.18	36559	2189
**CD55**	Soft tissue disorders	0.00	1.18	47409	4935
**CD55**	Conduction disorders	0.04	1.17	43104	2033
**CD55**	Arthrosis	0.00	1.16	42537	8167
**CD55**	Other and unspecified iron deficiency	0.04	1.16	49093	2312
**CD55**	Any mental disorder	0.00	1.15	48471	7722
**CD55**	Atrial fibrillation and flutter	0.01	1.15	30809	3662
**CD55**	Diseases of the respiratory system	0.00	1.15	45735	10129
**CD55**	Spondylopathies	0.03	1.15	45789	3002
**CD55**	Constipation	0.03	1.14	49229	3225
**CD55**	Coronary atherosclerosis	0.01	1.14	47089	3999
**CD55**	Diseases of the musculoskeletal system and connective tissue	0.00	1.14	42031	13980
**CD55**	Other gastritis (incl. Duodenitis)	0.02	1.14	41119	3875
**CD55**	Other cataract	0.03	1.13	46656	4182
**CD55**	Diaphragmatic hernia	0.03	1.12	44629	4295
**CD55**	Diseases of oesophagus, stomach and duodenum	0.01	1.10	45544	8300
**CD55**	Metabolic disorders	0.01	1.10	47469	9607
**CD55**	Neurological diseases	0.02	1.10	46880	7287
**GGCT**	Impacted teeth	0.01	1.64	44566	87

**GGCT**	Benign lipomatous neoplasm of skin and subcutaneous tissue of limbs	0.01	1.56	44554	105
**GGCT**	Bronchitis, not specified as acute or chronic	0.02	1.56	38190	99
**GGCT**	Epiphora	0.02	1.56	42974	95
**GGCT**	Myalgia	0.01	1.54	38294	131
**GGCT**	Embedded and impacted teeth	0.04	1.48	44554	92
**GGCT**	Seronegative rheumatoid arthritis	0.04	1.48	34974	103
**GGCT**	Crohn disease (strict definition, all UC cases excluded)	0.04	1.46	44083	107
**GGCT**	Disorders of phosphorus metabolism and phosphatases	0.00	1.46	34414	220
**GGCT**	Malignant neoplasm of stomach, excluding all cancers (controls excluding all cancers)	0.03	1.44	35640	129
**GGCT**	Crohn disease	0.02	1.43	41807	151
**GGCT**	Ulcerative colitis, NAS	0.01	1.41	41888	232
**GGCT**	Retinal detachment with retinal break	0.01	1.40	42767	190
**GGCT**	Postmenopausal atrophic vaginitis	0.05	1.38	18123	142
**GGCT**	Ulcerative colitis	0.01	1.37	41934	278
**GGCT**	Other specified disorders of kidney and ureter	0.01	1.35	44077	257
**GGCT**	Allergic asthma (mode)	0.03	1.34	30668	207
**GGCT**	Other specified and unspecified retinal disorders	0.04	1.32	42778	201
**GGCT**	Emphysema	0.00	1.31	38640	549
**GGCT**	Fibromyalgia	0.01	1.31	38554	391
**GGCT**	Chronic sinusitis	0.05	1.26	42457	266
**GGCT**	Sciatica+with lumbago	0.01	1.26	38959	433
**GGCT**	Bronchiectasis	0.02	1.22	38695	604
**GGCT**	Other/unspecified rheumatoid arthritis	0.01	1.19	44475	755
**GGCT**	Hypokalaemia	0.02	1.18	35027	833
**GGCT**	Other (seronegative) rheumatoid arthritis, wide	0.02	1.18	44455	776
**GGCT**	Rheumatoid arthritis	0.03	1.17	31763	787
**GGCT**	Diseases of peritoneum	0.03	1.16	44553	826
**GGCT**	Disorders of mineral metabolism	0.03	1.15	35144	950
**GGCT**	Other and unspecified iron deficiency	0.01	1.13	44158	1992
**GGCT**	Disorders of choroid and retina	0.02	1.12	44357	1780
**GGCT**	Chronic lower respiratory diseases	0.00	1.10	42933	4842
**GGCT**	Noninfective enteritis and colitis	0.04	1.10	43378	1722
**GGCT**	Other respiratory disorders and diseases	0.02	1.10	44516	2239
**GGCT**	Asthma, excluding more controls	0.02	1.09	33080	3081
**GGCT**	Asthma, unspecified (mode)	0.02	1.09	33493	3032
**GGCT**	Disorders of lipoprotein metabolism and other lipidaemias	0.00	1.09	40255	6061
**GGCT**	Other soft tissue disorders, not elsewhere classified	0.04	1.09	40288	2125
**GGCT**	Type 2 diabetes without complications	0.01	1.09	43640	3198
**GGCT**	Depression or dysthymia	0.04	1.08	39044	2597
**GGCT**	Metabolic disorders	0.00	1.08	42754	8560
**GGCT**	Other diseases of pleura	0.03	1.08	44293	3400
**GGCT**	Diseases of the respiratory system	0.00	1.07	41104	9024
**GGCT**	Polyarthropathies	0.05	1.06	35246	4270
**KRT17**	Coeliac disease	0.04	1.52	40528	95
**KRT17**	Other deformities of toe(s)	0.04	1.49	30136	120

**KRT17**	Bradycardia, unspecified, drug-induced	0.04	1.48	42634	103
**KRT17**	Dermatitis and eczema	0.03	1.40	42524	181
**KRT17**	Type 1 diabetes without complications	0.04	1.38	39361	180
**KRT17**	Postmenopausal bleeding	0.04	1.21	18223	781
**PSME2**	Other diseases of blood and blood-forming organs	0.00	2.68	49754	124
**PSME2**	Coeliac disease	0.00	2.64	47710	248
**PSME2**	Intestinal malabsorption	0.00	2.64	47749	287
**PSME2**	Lymphoid leukaemia	0.00	2.30	40533	102
**PSME2**	Systemic lupus erythematosus	0.00	2.20	49740	256
**PSME2**	Sicca syndrome [Sjšgren]	0.00	2.14	49665	181
**PSME2**	Crohn's disease NAS	0.00	2.13	47579	172
**PSME2**	Crohn disease	0.00	2.10	47590	183
**PSME2**	Other and unspecified types of non-Hodgkin lymphoma, hilmo, excluding all cancers	0.00	1.97	40624	193
**PSME2**	Sarcoidosis	0.00	1.97	50679	110
**PSME2**	Disorders of iron metabolism	0.00	1.94	38881	118
**PSME2**	Lupus erythematosus	0.00	1.93	46647	111
**PSME2**	Respiratory insufficiency	0.01	1.91	34746	105
**PSME2**	Crohn disease (strict definition, all UC cases excluded)	0.00	1.87	50184	135
**PSME2**	Disorders of plasma-protein metabolism, not elsewhere classified	0.00	1.85	38883	120
**PSME2**	Generalized epilepsy	0.01	1.82	50131	107
**PSME2**	Bacterial pneumonia, not elsewhere classified	0.00	1.80	46817	160
**PSME2**	Disorders of phosphorus metabolism and phosphatases	0.00	1.77	39014	251
**PSME2**	Recurrent and persistent haematuria	0.02	1.75	50395	97
**PSME2**	Small fibre neuropathy	0.02	1.73	50250	98
**PSME2**	Other/unspecified seropositive rheumatoid arthritis	0.02	1.72	50837	107
**PSME2**	Diseases of the myoneural junction and muscle	0.02	1.69	50848	121
**PSME2**	Acute peritonitis	0.00	1.67	49946	182
**PSME2**	Chronic lymphocytic leukaemia and small lyphocytis leukaemia, excluding all cancers (controls excluding all cancers)	0.03	1.67	40533	102
**PSME2**	Diseases of spleen	0.01	1.66	49770	140
**PSME2**	Postprocedural endocrine and metabolic disorders, not elsewhere classified	0.00	1.66	39091	328
**PSME2**	Implicit viral Sepsis	0.00	1.63	44461	780
**PSME2**	Alkalosis	0.04	1.61	38874	111
**PSME2**	Benign lipomatous neoplasm of skin and subcutaneous tissue of limbs	0.03	1.60	50708	114
**PSME2**	Other coagulation defects	0.00	1.60	50185	222
**PSME2**	Emphysema	0.00	1.58	44031	620
**PSME2**	Hyperkalaemia	0.00	1.58	39348	585
**PSME2**	Dialysis	0.02	1.55	45886	155
**PSME2**	Other psoriatic arthropathies	0.04	1.54	50843	134
**PSME2**	Psoriatic arthropathies	0.03	1.54	35360	137
**PSME2**	Bipolar affective disorders	0.04	1.53	47261	136
**PSME2**	Viral and other specified intestinal infections	0.01	1.53	47956	219
**PSME2**	Allergic asthma (mode)	0.01	1.52	34872	231
**PSME2**	Disorders of calcium metabolism	0.00	1.50	39311	548

**PSME2**	Arthropathic psoriasis	0.05	1.49	50241	140
**PSME2**	Bacterial infection, other or unspecified	0.01	1.48	48828	296
**PSME2**	Non-Hodgkin lymphoma, all, excluding all cancers (controls excluding all cancers)	0.00	1.48	40750	319
**PSME2**	Cardiac arrest	0.00	1.47	28212	378
**PSME2**	Disorders of mineral metabolism	0.00	1.47	39851	1088
**PSME2**	Hypokalaemia	0.00	1.47	39711	948
**PSME2**	Malignant neoplasm of kidney, except renal pelvis, excluding all cancers (controls excluding all cancers)	0.02	1.47	40644	213
**PSME2**	Other arterial embolism and thrombosis	0.02	1.47	48552	198
**PSME2**	Other specified and unspecified retinal disorders	0.01	1.47	48648	235
**PSME2**	Other/unspecified rheumatoid arthritis	0.00	1.47	50623	855
**PSME2**	Other specified disorders of kidney and ureter	0.01	1.46	50120	310
**PSME2**	Lung cancer and mesothelioma	0.00	1.45	35291	650
**PSME2**	Other (seronegative) rheumatoid arthritis, wide	0.00	1.45	50599	881
**PSME2**	Postmenopausal atrophic vaginitis	0.05	1.45	20622	159
**PSME2**	Secondary right heart disease	0.00	1.45	49653	664
**PSME2**	Myocardial infarction, with ST-elevation	0.01	1.44	50697	308
**PSME2**	Other and unspecified polyneuropathies, also in other diseases	0.00	1.44	50734	582
**PSME2**	Rheumatoid arthritis	0.00	1.44	36116	893
**PSME2**	Ulcer of lower limb, not elsewhere classified	0.00	1.44	47003	467
**PSME2**	Inguinal hernia, bilateral	0.04	1.43	41502	202
**PSME2**	Ulcerative colitis, NAS	0.02	1.43	47667	260
**PSME2**	Disorders of magnesium metabolism	0.01	1.42	39139	376
**PSME2**	Interstitial lung disease	0.00	1.42	48408	629
**PSME2**	Other pulmonary heart/vessel disease	0.00	1.42	49664	675
**PSME2**	Fluid overload	0.00	1.41	39268	505
**PSME2**	Implicit fungal Sepsis	0.00	1.41	44752	506
**PSME2**	Disorders of parathyroid gland	0.02	1.39	50351	274
**PSME2**	Other disorders of glucose regulation and pancreatic internal secretion	0.00	1.39	50857	439
**PSME2**	Bronchiectasis	0.00	1.38	44099	688
**PSME2**	Dementia in other diseases classified elsewhere	0.04	1.38	49081	252
**PSME2**	Hypertension, Pulmonary Arterial	0.01	1.38	33788	345
**PSME2**	Implicit bacterial Sepsis	0.00	1.38	45000	2177
**PSME2**	Hyperparathyroidism	0.04	1.37	50334	257
**PSME2**	Idiopathic pulmonary fibrosis	0.00	1.37	48341	562
**PSME2**	Ulcerative colitis	0.02	1.37	47722	315
**PSME2**	Acidosis	0.00	1.36	39385	622
**PSME2**	Other disorders of penis	0.04	1.36	19168	280
**PSME2**	Acute renal failure	0.00	1.35	48834	3103
**PSME2**	Non-small cell lung cancer, excluding all cancers (controls excluding all cancers)	0.01	1.35	40872	441
**PSME2**	Other respiratory diseases principally affecting the interstitium	0.00	1.35	50711	787
**PSME2**	Bacterial pneumonia (organism specified)	0.05	1.34	50076	271
**PSME2**	Coxarthrosis, primary	0.02	1.34	50614	372
**PSME2**	Hypertensive Renal Disease	0.05	1.34	33707	264

**PSME2**	Volume depletion	0.00	1.34	40277	1514
**PSME2**	Other disorders of fluid, electrolyte and acid-base balance	0.00	1.33	41882	3119
**PSME2**	Ventral hernia	0.01	1.32	41814	514
**PSME2**	Cellulitis	0.00	1.31	49627	1376
**PSME2**	Dental caries	0.02	1.31	49421	452
**PSME2**	Implicit Sepsis	0.00	1.30	35594	3910
**PSME2**	Other arrhythmias	0.00	1.30	42866	826
**PSME2**	Other disorders of bladder	0.00	1.30	45767	1415
**PSME2**	Cholecystitis	0.04	1.29	47293	399
**PSME2**	Migraine	0.01	1.29	47948	706
**PSME2**	Mycoses	0.00	1.29	50785	996
**PSME2**	Vitamin D deficiency	0.00	1.29	46413	722
**PSME2**	Other specified cerebrovascular diseases, other cerebrovascular disorders in diseases classified elsewhere	0.00	1.28	49138	1209
**PSME2**	Purpura and other haemorrhagic conditions	0.02	1.28	50508	545
**PSME2**	Type 2 diabetes without complications	0.00	1.28	49631	3628
**PSME2**	Diarrhoea and gastroenteritis of presumed infectious origin	0.00	1.27	50133	2396
**PSME2**	Other acute lower respiratory infections	0.00	1.27	50392	1905
**PSME2**	Unspecified acute lower respiratory infection	0.00	1.27	50348	1861
**PSME2**	Chronic kidney disease	0.00	1.26	48724	2993
**PSME2**	Early onset COPD	0.02	1.26	48761	617
**PSME2**	Epilepsy	0.02	1.26	47809	567
**PSME2**	Explicit Sepsis	0.00	1.26	46588	1922
**PSME2**	Hypo-osmolality and hyponatraemia	0.00	1.26	39950	1187
**PSME2**	Keratitis	0.03	1.26	50615	531
**PSME2**	Pneumonia, organism unspecified	0.00	1.26	49942	3285
**PSME2**	Streptococcus and staphylococcus as the cause of diseases classified to other chapters	0.01	1.26	48158	820
**PSME2**	Migraine, unspecified, drug-induced	0.02	1.25	50744	637
**PSME2**	Myocardial infarction, strict	0.00	1.25	45528	1397
**PSME2**	Other respiratory disorders and diseases	0.00	1.25	50672	2566
**PSME2**	Influenza and pneumonia	0.00	1.24	50437	3780
**PSME2**	Intestinal infectious diseases	0.00	1.24	50557	2820
**PSME2**	Other disorders of kidney and ureter	0.01	1.24	50750	940
**PSME2**	Other septicaemia	0.00	1.24	50387	1855
**PSME2**	Renal tubulo-interstitial diseases	0.00	1.24	50391	5257
**PSME2**	Candidiasis	0.01	1.23	50631	842
**PSME2**	Condition for Implicit Sepsis (Organ dysfunction codes)	0.00	1.23	49302	6326
**PSME2**	Diseases of peritoneum	0.01	1.23	50710	946
**PSME2**	Ischaemic Stroke, excluding all haemorrhages	0.01	1.23	50136	1102
**PSME2**	Osteoporosis	0.00	1.23	48969	2222
**PSME2**	Other bacterial diseases	0.00	1.23	50652	2120
**PSME2**	Other disorders of bone density and structure	0.02	1.23	47494	747
**PSME2**	Other or unspecified ileus, impaction or obstruction	0.03	1.23	36484	678
**PSME2**	Pleural effusion	0.00	1.23	48440	1943

**PSME2**	COPD, hospital admissions 1, only main diagnosis	0.00	1.22	50486	2342
**PSME2**	Delirium, not induced by alcohol and other psychoactive substances	0.01	1.22	49951	1122
**PSME2**	Nonalcoholic fatty liver disease	0.03	1.22	50824	722
**PSME2**	Cerebral palsy and other paralytic syndromes	0.05	1.21	50723	641
**PSME2**	Cholelithiasis	0.00	1.21	48852	1958
**PSME2**	Diseases of the digestive system	0.00	1.21	50264	2802
**PSME2**	Hypotension	0.00	1.21	50600	2333
**PSME2**	Other bacterial agents as the cause of diseases classified to other chapters	0.00	1.21	49164	1826
**PSME2**	Other disorders of urethra and urinary system	0.00	1.21	47793	3441
**PSME2**	Heart failure, strict	0.00	1.20	50480	2160
**PSME2**	Infections of the skin and subcutaneous tissue	0.00	1.20	50106	1855
**PSME2**	Other diseases of urinary system	0.00	1.20	48812	4460
**PSME2**	Rheumatic valve diseases	0.01	1.20	50742	1287
**PSME2**	Disorders of gallbladder, biliary tract and pancreas	0.00	1.19	49650	2756
**PSME2**	Later onset COPD	0.00	1.19	48914	1725
**PSME2**	Major coronary heart disease event	0.00	1.19	49930	1656
**PSME2**	Other diseases of pleura	0.00	1.19	50418	3921
**PSME2**	Other peripheral vascular diseases	0.02	1.19	49411	1057
**PSME2**	Other/unspecified dorsalgia	0.02	1.19	44958	1126
**PSME2**	Stroke, excluding SAH	0.01	1.19	50114	1266
**PSME2**	Stroke, including SAH	0.02	1.18	50073	1341
**PSME2**	Certain infectious and parasitic diseases	0.00	1.17	49094	6913
**PSME2**	Coronary atherosclerosis	0.00	1.17	48180	4084
**PSME2**	Other and unspecified iron deficiency	0.00	1.17	50242	2352
**PSME2**	Bacterial, viral and other infectious agents	0.00	1.16	50063	2725
**PSME2**	GI-bleeding	0.00	1.16	49754	2292
**PSME2**	Hyperlipidaemia, other/unspecified	0.03	1.16	40003	1240
**PSME2**	Other diseases of the digestive system	0.00	1.16	49783	2321
**PSME2**	Other specified/unspecified soft tissue disorders	0.04	1.16	44513	1086
**PSME2**	Diseases of the respiratory system	0.00	1.15	46829	10371
**PSME2**	Disorders of choroid and retina	0.01	1.15	50464	2051
**PSME2**	Metabolic disorders	0.00	1.15	48568	9805
**PSME2**	Osteopathies and chondropathies	0.00	1.15	50116	3369
**PSME2**	Chronic lower respiratory diseases	0.00	1.14	48897	5486
**PSME2**	Diseases of the blood and blood-forming organs and certain disorders involving the immune mechanism	0.00	1.14	49331	5710
**PSME2**	Other cataract	0.00	1.14	47730	4280
**PSME2**	Other or ill-defined heart diseases	0.02	1.14	44068	2028
**PSME2**	Other soft tissue disorders, not elsewhere classified	0.01	1.14	45882	2455
**PSME2**	Genitourinary diseases	0.00	1.13	42488	10580
**PSME2**	Inguinal hernia	0.04	1.13	43076	1776
**PSME2**	Ischaemic heart disease, wide definition	0.00	1.13	48517	4386
**PSME2**	Noninfective enteritis and colitis	0.03	1.13	49388	1981
**PSME2**	Smoking dependency	0.01	1.13	49836	2687
**PSME2**	Diseases of male genital organs	0.01	1.12	22167	3279
**PSME2**	Falls/tendency to fall	0.00	1.12	49708	5088

**PSME2**	Hyperplasia of prostate	0.02	1.12	21459	2571
**PSME2**	Anxiety disorders, excluding more controls	0.02	1.11	44528	2805
**PSME2**	Asthma, unspecified (mode)	0.02	1.11	38072	3431
**PSME2**	Episodal and paroxysmal disorders	0.03	1.11	49945	2703
**PSME2**	Other gastritis (incl. Duodenitis)	0.01	1.11	42057	3931
**PSME2**	Asthma, excluding more controls	0.02	1.10	37587	3482
**PSME2**	Disorders of lipoprotein metabolism and other lipidaemias	0.00	1.10	45721	6958
**PSME2**	Substance abuse, excluding more controls	0.04	1.10	44924	3201
**PSME2**	Diverticular disease of intestine	0.01	1.09	42015	6209
**PSME2**	Mood disorders, excluding more controls	0.05	1.09	44841	3118
**PSME2**	Death due to cardiac causes	0.00	1.08	44422	14883
**PSME2**	Gastrointestinal diseases	0.00	1.08	39410	16332
**PSME2**	Hypertension, essential	0.00	1.08	46266	12823
**PSME2**	Neurological diseases	0.01	1.08	47982	7428
**PSME2**	Any mental disorder	0.02	1.07	49594	7871
**PSME2**	Gastro-oesophageal reflux disease	0.05	1.07	43056	4930
**PSME2**	Hernia	0.02	1.07	47684	6384
**PSME2**	Hypertension	0.00	1.07	46232	12789
**PSME2**	Other diseases of intestines	0.01	1.06	46936	11130
**SERPINC1**	Chronic kidney disease	0.00	7.47	40637	81
**SERPINC1**	Diseases of pulp and periapical tissues	0.00	4.88	43266	175
**SERPINC1**	Malignant neoplasm of prostate, excluding all cancers (controls excluding all cancers)	0.00	4.54	16144	200
**SERPINC1**	Malignant neoplasm of colon, excluding all cancers (controls excluding all cancers)	0.02	4.13	35677	112
**SERPINC1**	Cerebral palsy and other paralytic syndromes	0.01	4.12	44244	145
**SERPINC1**	Inflammatory diseases of prostate (prostatitis)	0.03	4.11	16702	88
**SERPINC1**	Polyarthrosis	0.02	4.04	31129	121
**SERPINC1**	Soft tissue disorders related to use, overuse and pressure	0.04	3.97	38284	86
**SERPINC1**	Gout	0.02	3.88	31130	122
**SERPINC1**	Other deformities of toe(s)	0.02	3.86	31129	121
**SERPINC1**	Other pulmonary heart/vessel disease	0.01	3.78	43276	173
**SERPINC1**	Unspecified kidney failure	0.05	3.59	40657	101
**SERPINC1**	Secondary right heart disease	0.01	3.44	43274	171
**SERPINC1**	Benign lipomatous neoplasm of skin and subcutaneous tissue of limbs	0.02	3.42	44671	154
**SERPINC1**	Polycystic ovarian syndrome, consortium definition	0.02	3.26	24183	177
**SERPINC1**	Paralytic ileus and intestinal obstruction	0.03	2.87	31786	174
**SERPINC1**	Intestinal stricture	0.04	2.81	43937	174
**SERPINC1**	Coxarthrosis, primary	0.02	2.68	44445	253
**SERPINC1**	Hyperplasia of prostate	0.00	2.58	17103	489
**SERPINC1**	Epilepsy	0.03	2.43	41886	260
**SERPINC1**	Pure hypercholesterolaemia	0.00	2.32	35775	1545
**SERPINC1**	Mood disorders, excluding more controls	0.00	2.28	37318	548
**SERPINC1**	Type 2 diabetes without complications	0.00	2.25	41401	902
**SERPINC1**	Depression	0.01	2.22	41988	481
**SERPINC1**	Other arthrosis	0.00	2.20	31681	673

**SERPINC1**	Disorders of lipoprotein metabolism and other lipidaemias	0.00	2.18	35898	1668
**SERPINC1**	Type 2 diabetes	0.00	2.15	41426	927
**SERPINC1**	Other malignant neoplasms of skin (=non-melanoma skin cancer), excluding all cancers (controls excluding all cancers)	0.02	2.05	36022	457
**SERPINC1**	Squamous cell neoplasms and carcinomas of the skin, excluding all cancers (controls excluding all cancers)	0.02	2.01	36035	470
**SERPINC1**	Metabolic disorders	0.00	1.87	36194	1964
**SERPINC1**	Functional dyspepsia	0.01	1.85	34342	755
**SERPINC1**	Coxarthrosis	0.04	1.83	31508	500
**SERPINC1**	Diseases of male genital organs	0.00	1.81	17702	1088
**SERPINC1**	Arthrosis	0.00	1.79	33004	1996
**SERPINC1**	Meniscus derangement	0.02	1.79	31741	733
**SERPINC1**	Artrosis, including avohilmo	0.00	1.72	37496	1996
**SERPINC1**	Hypertension	0.00	1.68	33374	3872
**SERPINC1**	Hypertension, essential	0.00	1.64	33334	3832
**SERPINC1**	Gonarthrosis	0.04	1.58	31922	914
**SERPINC1**	Internal derangement of knee	0.04	1.58	31856	848
**SERPINC1**	Disorders of gallbladder, biliary tract and pancreas	0.04	1.51	42325	1063
**SERPINC1**	Other joint disorders	0.00	1.51	33269	2261
**SERPINC1**	Excessive, frequent and irregular menstruation	0.04	1.50	19206	1187
**SERPINC1**	Dorsopathies	0.02	1.49	40212	1637
**SERPINC1**	Other dorsopathies, not elsewhere classified	0.03	1.47	39871	1296
**SERPINC1**	Diaphragmatic hernia	0.03	1.44	37881	1511
**SERPINC1**	Hernia	0.01	1.40	39206	2836
**SERPINC1**	Arthropathies	0.01	1.34	35059	4051
**SERPINC1**	Neurological diseases	0.04	1.32	38478	2569
**SERPINC1**	Diseases of the musculoskeletal system and connective tissue	0.02	1.23	32237	6962
**SERPINC1**	Gastrointestinal diseases	0.02	1.20	30486	10120

Table S6. Proteins Showed Significant Differences after Eight Weeks Treatment in High Bleeders (2 Months vs. Baseline)

ProteinID	Gene Name	Fold Change	*P* value	Adjusted *p* value	Disease Marker
A0A0C4DH35	IGHV3-35	-5.39	0.000	0.008	
A0A0C4DH38	IGHV5-51	-5.21	0.000	0.008	
P07900	HSP90AA1	-4.96	<0.0001	0.000	
A0A0C4DH72	IGKV1-6	-4.47	0.001	0.016	
Q6ZN66	GBP6	-4.38	<0.0001	0.007	Yes
Q969H8	MYDGF	-4.23	<0.0001	0.001	Yes
P05109	S100A8	-4.03	<0.0001	0.001	
P00352	ALDH1A1	-3.92	0.000	0.011	Yes
P49643	PRIM2	-3.89	0.007	0.070	
P06702	S100A9	-3.86	<0.0001	0.001	
P04259	KRT6B	-3.81	0.003	0.048	
A0A0C4DH36	IGHV3-38	-3.23	0.000	0.011	
Q8NFT8	DNER	-3.16	<0.0001	0.003	Yes

P80511	S100A12	-3.14	0.001	0.016	Yes
Q93077	H2AC6	-3.14	0.008	0.084	
Q86T26	TMPRSS11B	-3.10	<0.0001	0.003	Yes
Q9BQR3	PRSS27	-3.03	0.003	0.047	Yes
O43278	SPINT1	-2.99	<0.0001	0.003	Yes
P68133	ACTA1	-2.89	0.031	0.189	
P53004	BLVRA	-2.81	0.000	0.011	
Q15365	PCBP1	-2.81	<0.0001	0.001	
P16403	H1-2	-2.79	0.004	0.057	
P17900	GM2A	-2.71	0.000	0.011	Yes
Q86VR7	VSIG10L	-2.71	0.006	0.069	Yes
Q04695	KRT17	-2.68	0.001	0.019	Yes
P27348	YWHAQ	-2.58	0.000	0.008	Yes
P08174	CD55	-2.51	0.005	0.057	Yes
P58546	MTPN	-2.51	0.006	0.068	Yes
Q04917	YWHAH	-2.45	0.005	0.060	
P28676	GCA	-2.36	0.003	0.047	
Q9BPY8	HOPX	-2.35	0.007	0.074	
P00734	F2	-2.33	0.004	0.057	Yes
A0A0A0MT36	IGKV6D-21	-2.28	0.025	0.170	
O00764	PDXK	-2.28	0.001	0.019	
P14625	HSP90B1	-2.28	0.008	0.079	Yes
Q13231	CHIT1	-2.28	0.020	0.145	Yes
P39687	ANP32A	-2.23	0.004	0.057	
P26583	HMGB2	-2.22	0.008	0.078	
P30050	RPL12	-2.17	0.017	0.128	
Q13404	UBE2V1	-2.16	0.012	0.101	
P68371	TUBB4B	-2.14	0.009	0.088	
Q9UL46	PSME2	-2.13	0.011	0.101	Yes
P06576	ATP5F1B	-2.10	0.015	0.117	
P27695	APEX1	-2.10	0.002	0.034	Yes
Q6ZMR5	TMPRSS11A	-2.10	0.005	0.060	
P06454	PTMA	-2.08	0.026	0.171	
P01008	SERPINC1	-2.04	0.002	0.038	Yes
P30520	ADSS2	-2.04	0.002	0.033	
P49189	ALDH9A1	-2.04	0.013	0.108	
P38159	RBMX	-1.99	0.013	0.110	
O75223	GGCT	-1.95	0.014	0.112	Yes
A0M8Q6	IGLC7	-1.91	0.050	0.266	
P20618	PSMB1	-1.89	0.045	0.249	
P08582	MELTF	-1.88	0.000	0.011	Yes
Q6XQN6	NAPRT	-1.88	0.005	0.060	Yes
P14550	AKR1A1	-1.80	0.041	0.237	
O75874	IDH1	-1.77	0.003	0.042	
P08865	RPSA	-1.75	0.023	0.164	
P19957	PI3	-1.75	0.003	0.041	Yes
P25788	PSMA3	-1.74	0.028	0.178	

P34932	HSPA4	-1.72	0.026	0.170	
P02763	ORM1	-1.71	0.002	0.038	Yes
P17174	GOT1	-1.69	0.014	0.113	Yes
P80748	IGLV3-21	-1.66	0.041	0.237	
P14324	FDPS	-1.61	0.025	0.170	
P61019	RAB2A	-1.58	0.038	0.226	
O60218	AKR1B10	-1.57	0.005	0.061	Yes
P05120	SERPINB2	-1.54	0.046	0.249	
Q9BRA2	TXNDC17	-1.51	0.042	0.240	
Q96DA0	ZG16B	1.65	0.000	0.008	
P27824	CANX	1.88	0.012	0.103	
P04264	KRT1	1.93	0.041	0.237	
A0A0B4J1V0	IGHV3-15	2.30	0.018	0.137	
P15328	FOLR1	2.55	0.012	0.101	Yes
P28072	PSMB6	2.60	0.001	0.013	
A0A0G2JRQ6		2.91	0.010	0.099	
P15516	HTN3	3.16	0.005	0.060	
P0DP25	CALM3	3.63	0.001	0.013	
Q9UKR0	KLK12	4.82	0.000	0.011	Yes
Q9BYB0	SHANK3	8.06	0.001	0.013	

Table S7. GO Biological Process Showed Significant Differences After 8 Weeks Treatment in High Bleeders

Downregulated in High Bleeders (8 Weeks vs. Baseline)		
GO Biological Process	FDR *P* Value	Genes
Cellular detoxification	8.54E-03	AKR1B10 AKR1A1 TXNDC17 S100A9 ALDH1A1
Cellular response to toxic substance	9.28E-03	AKR1B10 AKR1A1 TXNDC17 S100A9 ALDH1A1
Proteolysis	9.30E-03	PSMB1 PSMA3 PSME2 SERPINC1 HSP90B1 SERPINB2 S100A8 RBMX S100A9 MELTF F2 PI3 SPINT1 PRSS27 TMPRSS11B TMPRSS11A
Defense response to bacterium	9.30E-03	PI3 GBP6 HMGB2 F2 S100A12 S100A8 S100A9
Antimicrobial humoral response	2.91E-02	PI3 S100A9 S100A12 F2
Innate Immune System	1.07E-07	PSMB1 HSP90AA1 PSMA3 PSME2 GCA PI3 CHIT1 IDH1 S100A8 NAPRT PDXK S100A9 S100A12 HSP90B1 F2 TUBB4B CD55 GM2A ORM1 UBE2V1
Cellular responses to stress	1.52E-05	PSMB1 MYDGF HSP90AA1 PSMA3 PSME2 BLVRA IDH1 HSP90B1 RPSA HSPA4 H2AC6 H1-2 TUBB4B RPL12
Immune System	1.03E-04	PSMB1 HSP90AA1 PSMA3 PSME2 GCA PI3 CHIT1 IDH1 S100A8 NAPRT PDXK S100A9 S100A12 HSP90B1 F2 GBP6 TUBB4B CD55 GM2A SERPINB2 ORM1 UBE2V1
