# Supplementary figures and images for: Multi-Omics Insights into Gingivitis from a Clinical Trial: Understanding the Role of Bacterial and Host Factors
Source: Microorganisms. 2025 Oct 15;13(10):2371. doi: 10.3390/microorganisms13102371 (PMC12566121; doi:10.3390/microorganisms13102371)

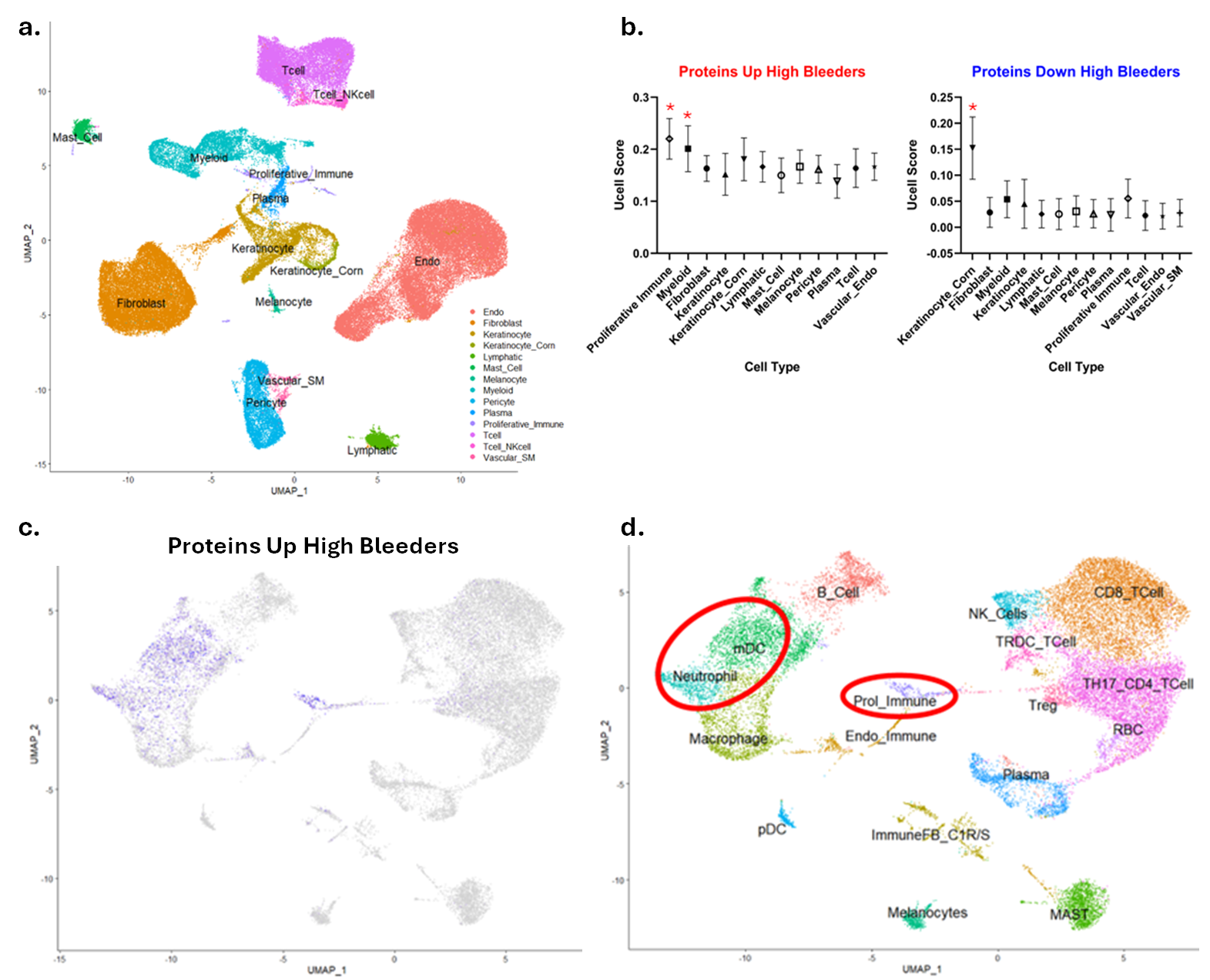

Supplement: Supplementary file 1 [file microorganisms-13-02371-s001.zip › Figure S1 Oct 13 2025.png]

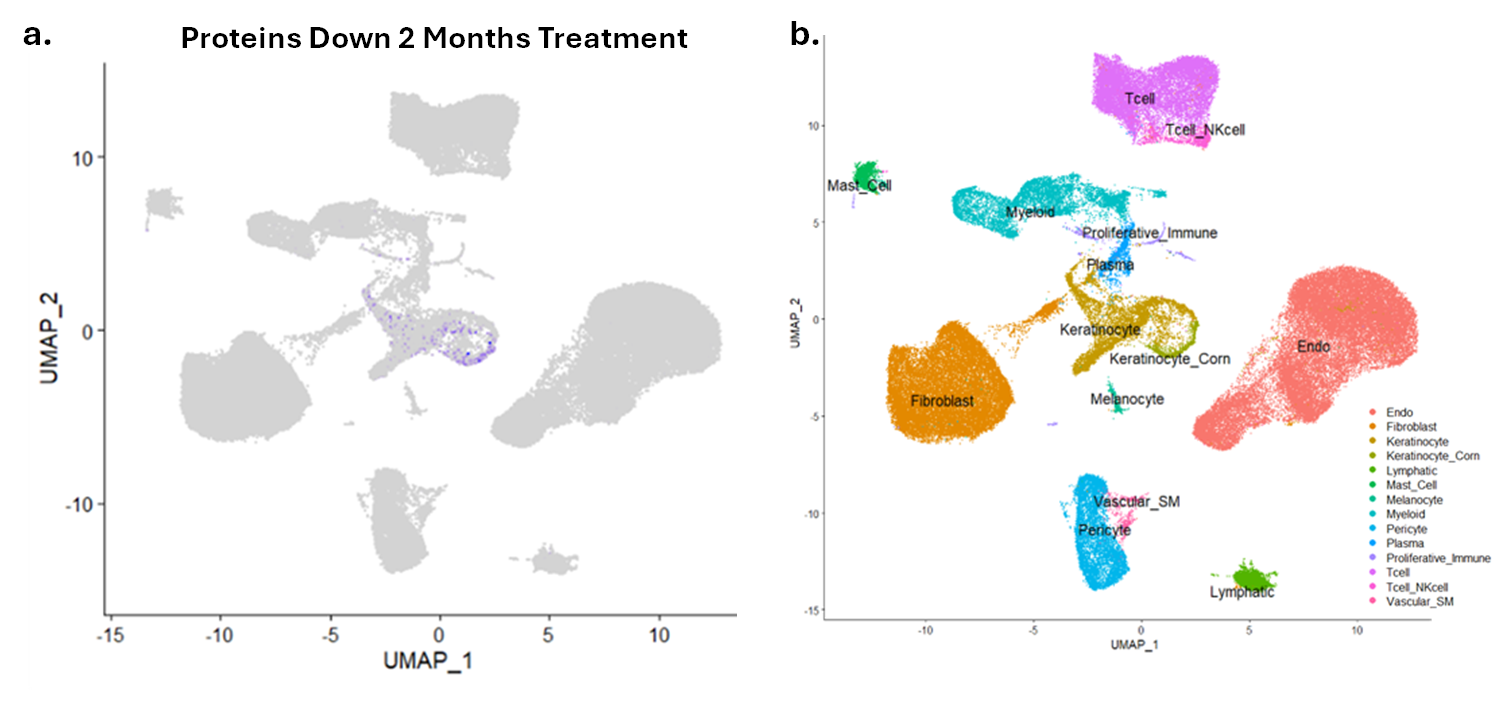

Supplement: Supplementary file 1 [file microorganisms-13-02371-s001.zip › Figure S2 Oct 13 2025.png]
